# Genome-wide identification, characterization, interaction network and expression profile of *GAPDH* gene family in sweet orange (*Citrus sinensis*)

**DOI:** 10.7717/peerj.7934

**Published:** 2019-11-14

**Authors:** Luke Miao, Chunli Chen, Li Yao, Jaclyn Tran, Hua Zhang

**Affiliations:** 1 Huazhong Agricultural University, College of Life Science and Technology, Wuhan, China; 2 Huazhong Agricultural University, Key Laboratory of Horticultural Plant Biology (Ministry of Education), Wuhan, China; 3 The University of Texas at Austin, Institute for Cellular and Molecular Biology, Austin, TX, USA; 4 The University of Texas at Austin, Department of Molecular Biosciences, Austin, TX, USA; 5 Huazhong Agricultural University, College of Resources and Environment, Wuhan, China

**Keywords:** *CsGAPDH*, Subcellular localization, Expression profile, Cis-element analysis, Protein–protein interact

## Abstract

Glyceraldehyde-3-phosphate dehydrogenase (GAPDH) is a key glycolytic enzyme that plays important roles in multiple cellular processes including phytohormone signaling, plant development, and transcriptional regulation. Although *GAPDH* genes have been well characterized in various plant species such as *Arabidopsis*, tobacco, wheat, rice, and watermelon, comprehensive analysis has yet to be completed at the whole genome level in sweet orange (*Citrus sinensis*). In this study, six *GAPDH* genes distributed across four chromosomes were identified within the sweet orange genome. Their gene structures, conserved subunits, and subcellular localization were also characterized. Cis-element analysis of *CsGAPDHs’* promoter regions and the results of dark treatments indicate that CsGAPDH may be involved in photosynthesis. *CsGAPDH* genes expressed either in a tissue-specific manner or constitutively were ultimately identified along with their expression response to phosphorus deficiency treatments. In addition, a dual-luciferase transient assay was performed to reveal the transcriptional activation of CsGAPDH proteins. Gene Ontology (GO) analysis for proteins interacting with CsGAPDHs helped to uncover the roles these CsGAPDHs play in other plant processes such as citrus seed germination. This study provides a systematic analysis of the *CsGAPDH* gene family in the sweet orange genome, which can serve as a strong foundation for further research into the biochemical properties and physiological functions of *CsGAPDHs*.

## Introduction

Glyceraldehyde-3-phosphate dehydrogenase (GAPDH) is best known for its glycolytic role of converting glyceraldehyde-3-phosphate into 1,3-bisphosphoglycerate (Sirover, 2011), but previous studies have begun to discover some of GAPDH’s additional functions. GAPDHs play key roles in diverse cellular processes, including autophagy (Colell et al., 2007; Fengsrud et al., 2000), mRNA regulation (Garcin, 2019), gene expression regulation (Zhang, Zhao & Zhou, 2017), immune response (Henry et al., 2015), redox sensing (Schneider et al., 2018; Schuppe-Koistinen et al., 1994), and even photosynthesis (Howard, Lloyd & Raines, 2011). To function within these cellular processes, previous studies have demonstrated GAPDHs interacting with various protein partners (Moparthi et al., 2015; Yang et al., 2018), alongwith diverse subcellular localization within the cytoplasm, nucleus, membrane, mitochondria (Sirover, 2012; Tristan et al., 2011), and chloroplasts (Howard, Lloyd & Raines, 2011).

GAPDH’s sequence is highly conserved across many organisms, yet only one isoform has been identified in animal cells. This isoform is responsible for many non-glycolytic roles and contains multiple subcellular locales and post-translational modifications (Sirover, 2018; Tristan et al., 2011). Subcellular localization appears to influence its subsequent functions: cytoplasmic GAPDHs are involved in mRNA stability and translation (Garcin, 2019) while nuclear GAPDHs have exhibited diverse roles in DNA repair, maintenance of DNA integrity (Ferreira et al., 2015; Kosova, Khodyreva & Lavrik, 2017), transcriptional regulation (Zhang, Zhao & Zhou, 2017) and histone biosynthesis (Zheng, Roeder & Luo, 2003).

As compared to animal cells’ singular isoform, plant cells have been shown to contain multiple GAPDH isoforms involved primarily with glycolytic or photosynthetic pathways with varying subcellular localization. For example, *Arabidopsis* plants possess seven isoforms: two cytosolic isoforms GAPC1/GAPC2 (Vescovi et al., 2013), three chloroplastic isoforms GAPA1/GAPA2/GAPB (Marri et al., 2005), and two plastidic isoforms GAPCp1/GAPCp2 (Anoman et al., 2015). Cytosolic isoforms catalyze the conversion of glyceraldehyde-3-phosphate to 1,3-bisphosphoglycerate in cytoplasm, while the chloroplastic isoforms are involved in the Calvin–Benson cycle by catalyzing the reduction of 1,3-diphosphoglycerate (Howard, Lloyd & Raines, 2011), and plastidic isoforms could be an important metabolic connector of glycolysis with other pathways, such as the phosphorylated pathway of serine biosynthesis, or the metabolism of γ-aminobutyrate, which in turn affect plant development (Anoman et al., 2015; Muñoz-Bertomeu et al., 2009; Petersen, Brinkmann & Cerff, 2003). GAPDH structure is best described as a tetramer conformation comprised of identical or highly similar subunits. Glycolytic GAPDHs (A4-GAPDH) have been shown to be comprised of identical subunits, while some photosynthetic isoforms (A2B2-GAPDH) contain highly similar subunits (Fermani et al., 2007; Zaffagnini et al., 2013). In addition, subunits of GAPDH tetramers are highly conserved, containing a Gp_dh_N domain (NAD(P)-binding domain) and a Gp_dh_C domain (catalytic domain) (Zeng et al., 2016). Many of the conserved amino acids displayed modifications such as acetylation, phosphorylation, succinylation or ubiquitination (Meng et al., 2018). However, the members of Citrus *GAPDH* gene family (*CsGAPDH* genes) have not been analyzed in detail with regard to their structures or functions.

As stated above, GAPDHs have been shown to function within other processes besides glycolysis and are especially prominent in developmental and stress response pathways. *Arabidopsis* plastidial GAPDHs are important in balancing sugar and amino acid levels necessary for root development (Muñoz-Bertomeu et al., 2009) and disruption of these GAPDHs can result in male sterility (Muñoz-Bertomeu et al., 2010). Plastidial GAPDHs are also involved in abscisic acid signal transduction, affecting seed germination and overall plant growth (Muñoz-Bertomeu et al., 2011). Within stress response pathways, NAD-dependent GAPDHs are key regulators in promoting growth of *Arabidopsis* seedlings under low selenate conditions (Takeda & Fukui, 2015). In roots, the cytoplasmic GADPH isoform GAPC1 accumulates within the nucleus in response to cadmium stress (Vescovi et al., 2013) and bacterial flagellin threats (Henry et al., 2015). The expression levels and enzymatic activity of GAPDHs can change dramatically during immune response (Henry et al., 2015). Although *Arabidopsis* GAPDHs are well characterized, the roles of Citrus *GAPDH* genes within development and stress response remain largely unknown.

Citrus is the world’s largest economic fruit crop and is grown across the world. *Citrus sinensis*, or sweet orange, is a variety of citrus that is well received by people due to its high economic and nutritional value. As production increases, pressure from both biotic and abiotic stress such as Huanglongbing, drought, or phosphorous deficiency, have challenged the plants’ tolerance to stress. GAPDHs have been shown to hold important roles within citrus stress resistance pathways including response to recurring water deficit or CTV (*Citrus tristeza virus*) virus infection. Given the importance of *GAPDHs* in stress and disease resistance alongside the inadequate information published on the identification and functional characterization of GAPDH proteins in *C. sinensis*, detailed and comprehensive analysis of the *GAPDH* gene family in the whole genome of sweet orange are presented in this study.

Here, six putative *CsGAPDH* genes were isolated and their phylogenetic relationships, chromosome distribution, gene expression patterns and cis-elements within their promoter regions were analyzed. In addition, the different gene expression patterns of each *CsGAPDH* gene under dark treatment and phosphorus deficiency were also examined. This study aims to provide an in-depth look at the *GAPDH* gene family and help to facilitate further functional characterization of *GAPDH* genes in citrus.

## Materials and Methods

### Citrus *GAPDH* gene identification and chromosome mapping in sweet orange

The method to identify all the putative *GAPDH* genes in sweet orange is derived from Zhang et al. (2019). Annotated proteins sequence were downloaded from the *C. sinensis* annotation project database of Huazhong Agricultural University (http://citrus.hzau.edu.cn/orange/) (Xu et al., 2013), phytozome Cirus sinensis v1.1 database (http://phytozome.jgi.doe.gov/pz/portal.html) (Wu et al., 2014), and the Citrus Genome Database (https://www.citrusgenomedb.org/). Then potential candidates were screened based upon homologous alignment among rice, *Arabidopsis* and sweet orange. The integrity of the Gp_dh_N and Gp_dh_C domain candidate genes were identified using SMART software with *e*-value < 0.1 (http://smart.embl-heidelberg.de/). Finally, the correctly predicted genes of sweet orange were named from *CsGAPDH1* to *CsGAPDH6* according to their phylogenetic relationships with *Arabidopsis*. Five *CsGAPDH* genes were mapped onto the chromosomes, and the schematic diagram distribution on the chromosomes was drawn using Photoshop software.

### Sequence alignment, phylogenetic analysis, and classification of *GAPDH* genes in *C. sinensis*

GAPDH protein sequences of *Arabidopsis* and Citrus species (https://www.arabidopsis.org/) were aligned using ClustalW program of MEGA 5.1 software. After alignment, GeneDoc was used to display the differential amino acids in the alignment results with default parameters. To classify and analyze the evolutionary relationship of GAPDHs identified from *C. sinensis*, related genus, and *Arabidopsis*, the phylogenetic analysis based on sequence alignments was conducted with MEGA 5.1 software using the Neighbor-joining statistical method and the bootstrap test carried out with 1,000 replications.

### Putative cis-acting regulatory elements and protein–protein interaction network of CsGAPDH proteins

The up-stream 1,500 bp sequence from the DNA transcription start site was used to analyze the cis-elements. Putative stress or hormone-responsive cis-elements located in the promoter region of *CsGAPDH* genes were obtained using PlantCARE online (http://bioinformatics.psb.ugent.be/webtools/plantcare/html/). The interacting proteins of CsGAPDHs were searched, employing ortholog-based and domain-based methods from the Orange Genome Annotation Project (http://citrus.hzau.edu.cn/orange/ppi/index.php) (Ding et al., 2014; Xu et al., 2013).

### GO analysis

Homologous genes of *CsGAPDHs* interacting genes in *Arabidopsis* were taken from the sweet orange annotation project database of Huazhong Agricultural University (http://citrus.hzau.edu.cn/orange/), and then GO analysis for the homologous genes were performed using the agriGO online database (http://bioinfo.cau.edu.cn/agriGO/analysis.php) (Tian et al., 2017).

### Seeds germination

Mature seeds, with their seed coat stripped off, were spread evenly in a petri dish containing two layers of filter paper and cultured in darkness at 28 °C. Replenish water in time in the process of culture to ensure the normal germination of seedlings. Three seeds were each taken at 0, 3, 6 and 9 days following germination and were immediately added to liquid nitrogen for RNA extraction.

### Plant growth condition and treatments with darkness in *C. sinensis* and phosphorus deficiency in *Poncirus trifoliata*

All the citrus seedlings studied were rooted from seeds. The seeds were spread in a petri dish containing moist filter paper and cultured at 28 °C in the dark. When the root length reached 2 cm, the seedlings were transferred into soil to grow and were cultivated in a greenhouse at 28 °C under 14 h-light/10 h-dark growth conditions. Consistent and robust plants were selected after a month of growth to be used in dark treatments to determine *CsGAPDH* gene expression. Fresh leaves were harvested after dark treatment of 12 h, 24 h, and 48 h respectively and stored in liquid nitrogen immediately. Plants grown under normal photoperiods were used as controls. For phosphorus deficiency treatment, 2-month-old *Poncirus trifoliata* (Pt) seedlings were irrigated with Hoagland solutions containing 1 μM P, while the controls were irrigated with 1 mM P (Zhang et al., 2019). Detection of *CsGAPDH* transcriptional levels in roots were measured after 1 week and 4 weeks of starvation treatment.

### RNA isolation and qRT-PCR

Total RNA was isolated from each sample using TransZol reagent (TransGen Biotech) according to the manufacturer’s instructions. RNA integrity and concentration were measured using 1% agarose gels and the NanoDrop 2000 (Thermo Scientific, Waltham, MA, USA) respectively. Four micrograms of total RNA was reverse-transcribed in a reaction of 20 μl, using TransCript One-Step gDNA Removal and cDNA Synthesis Super Mix Kit (TransGen Biotech, Beijing, China) to obtain first-strand cDNA. Melting curve analysis was utilized for detecting primer specificity before quantification with the following program: 95 °C for 15 s, 60 °C for 1 min, 95 °C for 15 s, 60 °C for 15 s. The quantitative PCR reaction procedure was performed on an Applied Biosystems^®^ QuantStudioTM 7 Flex Real-Time PCR System (Life Technologies, Carlsbad, CA, USA) at 95 °C for 10 min, 35 cycles of 95 °C for 15 s and 60 °C for 1 min. The relative expression levels were analyzed using the 2^−∆∆CT^ method. The *C. sinensis* Actin gene was used as the internal reference for normalization. All the primers for quantitative real-time PCR are listed in Table S1 and were designed using primer-blast (https://www.ncbi.nlm.nih.gov/tools/primer-blast/).

### Subcellular localization analysis

Constructed expression vector pFGC5941-CsGAPDHs-GFP was introduced into *Agrobacteriumtumefaciens* EHA105, and then the suspensions of Agrobacteria were infiltrated into tobacco leaves (*Nicotiana benthamiana*). Next, the plants were incubated in a culture room for 2 days. CsGAPDHs-GFP was observed by a Confocal Laser Scanning Microscopy. GFP was excited at 488 nm with an argon laser and fluorescence was detected by 505–550 nm band-pass filter. Band-pass filter 575–615 IR was used to detect chlorophyl auto-fluorescence.

### Transcriptional analysis of CsGAPDHs in protoplasts

In order to examine the transcriptional activity of CsGAPDH1/CsGAPDH2, full-length CsGAPDH1/CsGAPDH2 was fuzed to the GAL4 DNA binding domain to generate a GAL4DBD-CsGAPDH1/CsGAPDH2 fusion construct driven by the CaMV 35S promoter as an effector. 4×GAL4 was fuzed to luciferase driven by the CaMV 35S promoter as the reporter. A construct containing the Renilla luciferase gene driven by the *Arabidopsis* UBIQUITIN3 promoter was used as the internal control. The effector and reporter, and internal control, were co-transfected into *Arabidopsis* protoplasts in a ratio of 6:6:1 (effector:reporter:reference) respectively, and was then cultured for 12 h at 25 °C in darkness. The luciferase activities were measured with the Dual Luciferase Reporter Assay System (Promega, Madison, WI, USA).

## Results

### Identification and phylogenetic analysis of CsGAPDH proteins

**Figure 1:**
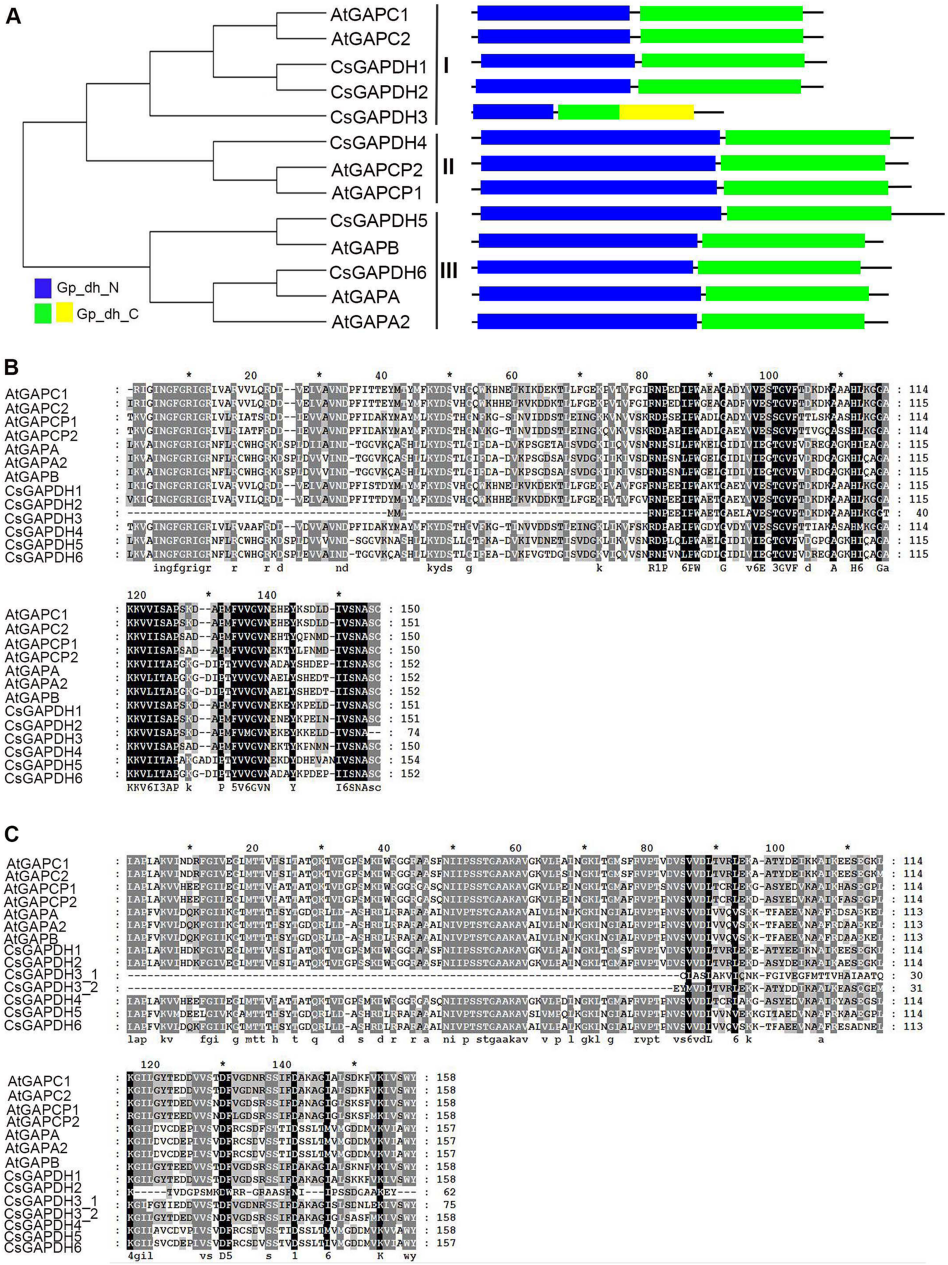
The phylogenetic analysis and multiple sequence alignment of CsGAPDH proteins. (A) Neighbour-joining phylogenetic tree of the CsGAPDH family. The phylogenetic tree was generated using the MEGA 5.1 software. In the schematic structure the predicted Gp_dh_N domain is in blue, the Gp_dh_C domain is in green and yellow. (B–C) Multiple sequence alignment of the Gp_dh_N and Gp_dh_C domain in Citrus and *Arabidopsis*. Fully and partially conserved residues are highlighted in black and gray boxes, respectively. Gaps (marked with dashes) have been introduced to maximize the alignments.

In order to run a complete search identifying *GAPDH* genes in the genome of sweet orange, all annotated proteins of the genome from the sweet orange annotation project database of Huazhong Agricultural University (http://citrus.hzau.edu.cn/orange/) (Xu et al., 2013), phytozome *C. sinensis* v1.1 datebase (https://phytozome.jgi.doe.gov/pz/portal.html) (Wu et al., 2014) and the Citrus Genome Database (https://www.citrusgenomedb.org/) were considered for analysis. After determining the integrity of the GAPDH protein domains using the online program SMART (http://smart.embl-heidelberg.de/) alongside sequence alignments with known *Arabidopsis* GAPDH protein sequences, 46 GAPDH proteins were identified in six species (clementine mandarin, pummelo, citron, papeda, sweet orange, and Chinese box orange; Data S1). Considering *C. sinensis* is the most cultivated variety of citrus with the most extensive ongoing research, the 12 CsGAPDH proteins identified from this species were the focus of this study. Protein sequences identified from two different genomic reference databases were largely duplicated (Fig. S1), resulting in six GAPDHs that were finally identified in *C. sinensis*. The detailed information of the *GAPDH* genes are listed in Table S2. In order to study the evolutionary relationships of the *GAPDH* gene family, a phylogenetic tree was built from the alignment of all six full-length GAPDH protein sequences in sweet orange and seven GAPDH protein sequences in *Arabidopsis* (Fig. 1A). *CsGAPDH* genes were designated *CsGAPDH1*, *CsGAPDH2*, *CsGAPDH3*, *CsGAPDH4*, *CsGAPDH5* and *CsGAPDH6* according to their phylogenetic relationship with *Arabidopsis*, which could further be clustered into three subgroups, subgroup I, II, and III (Fig. 1A). Proteins in the same subgroup may perform similar functions. Subgroup I corresponds with *Arabidopsis* GAPC proteins (CsGAPDH1, CsGAPDH2 and CsGAPDH3), subgroup II with GAPCp proteins (CsGAPDH4), and subgroup III with GAPA/B proteins (CsGAPDH5 and CsGAPDH6).

To examine the conserved amino acids of Gp_dh_N and Gp_dh_C subunits, multiple sequence alignments of both subunits in *Arabidopsis* and sweet orange were performed. All of the CsGAPDH proteins, excluding CsGAPDH3, showed highly conserved Gp_dh_N and Gp_dh_C subunits (Figs. 1A–1C). Moreover, two Gp_dh_C subunits were detected in CsGAPDH3 protein, both of which were much shorter than Gp_dh_C subunits found in other CsGAPDH proteins, indicating that CsGAPDH3 may have a distinct origin and function from the other CsGAPDH proteins.

### Chromosomal distribution of *CsGAPDH* genes

**Figure 2:**
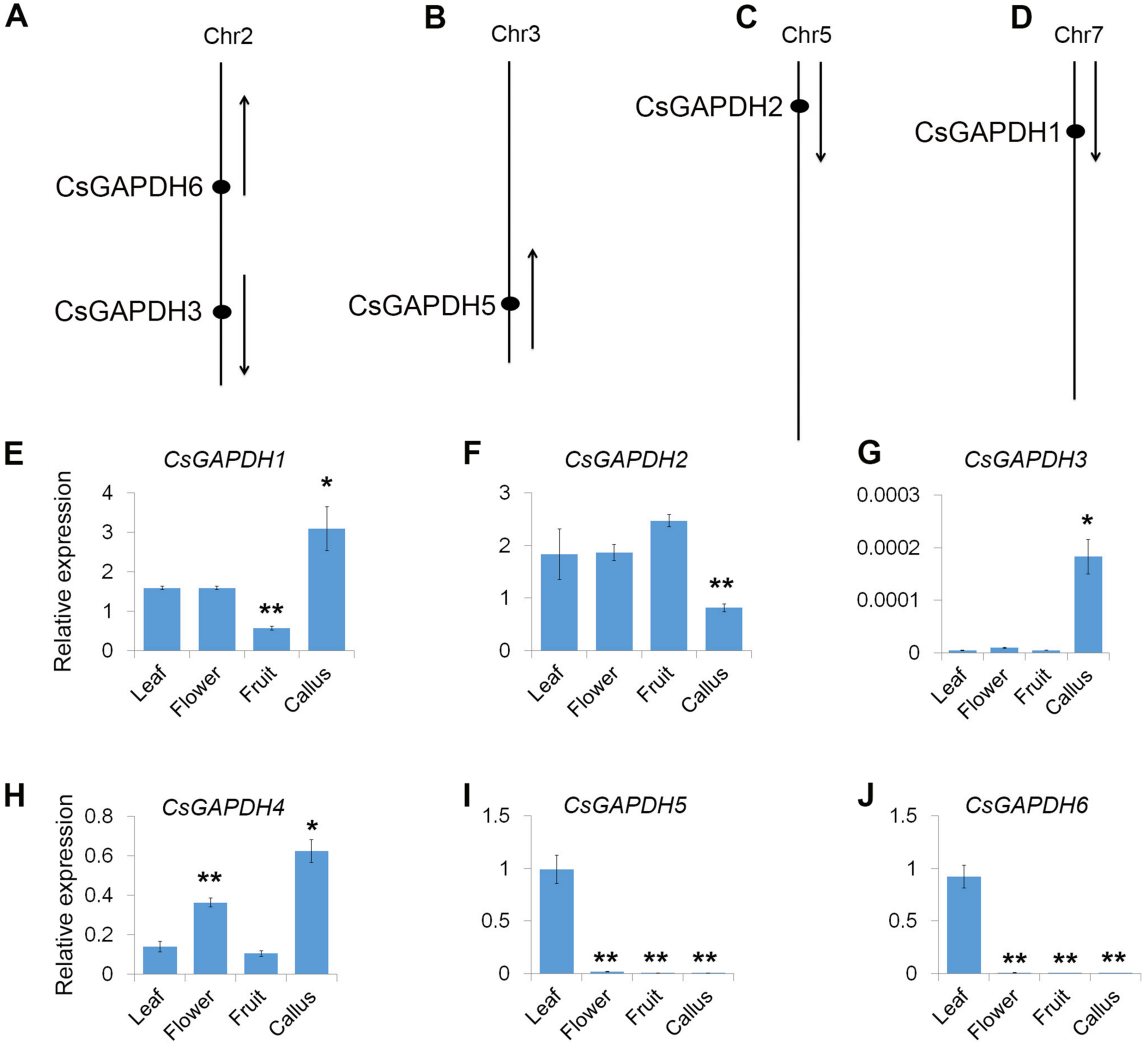
Genomic distribution and expression patterns of *CsGAPDH* genes. (A–D) Arrows next to gene names show the direction of transcription. Chromosome number is indicated at the top of each chromosome; (E–J) qPCR analysis expression of *CsGAPDH* genes in four tissues of sweet orange, including flower, leaf, fruit and callus. Error bars denotes the standard deviation calculated from three independent experiments. Statistical significance was analyzed by Student’s *t*-test (**p < 0.01, *p < 0.05).

The physical positions of the *GAPDH* genes were obtained from Huazhong Agricultural University’s sweet orange annotation database (http://citrus.hzau.edu.cn/orange/). The position and transcriptional direction of each gene are shown in Figs. 2A–2D, and the exact positions on *C. sinensis* chromosome pseudomolecules are given in Table S2. The data shows that five *CsGAPDH* genes were found to be dispersed across four chromosomes. *CsGAPDH1*, C*sGAPDH2*, and *CsGAPDH5* were localized on chromosome 7, chromosome 5, and chromosome 3, respectively, while both *CsGAPDH3* and *CsGAPDH6* were located on chromosome 2. There was no information on the position of *CsGAPDH4* within the sweet orange annotation database.

### Expression profiling of *CsGAPDH* genes

To reveal the transcriptional accumulation of *CsGAPDH* genes, the expression of six *CsGAPDH* genes were examined by real-time PCR in leaf, flower, fruit and callus tissues of the sweet orange (Figs. 2E–2J). Notably, transcriptional accumulation of *CsGAPDH* genes was found to demonstrate tissue-specific expression. *CsGAPDH3* showed extremely low expression levels in the tissues analyzed compared with the other five *CsGAPDH* genes, except for in calluses where it demonstrated a significantly higher expression level (Fig. 2G), indicating that it might be involved in the development of undifferentiated tissues/cells. In contrast, *CsGAPDH* genes in subgroup III (*CsGAPDH5* and *CsGAPDH6*) showed extremely high transcript accumulations in leaf tissue, but were much lower in the other three tissues (Figs. 2I and 2J). Therefore, suggesting that these genes might play roles in differentiated tissues/cells, especially in green tissues/cells. The other *CsGAPDH* genes were expressed constitutively and were found to be highly enriched in most of the tissues. *CsGAPDH1* showed a higher transcription level in callus tissues than in other three, while *CsGAPDH2* illustrated the opposite pattern (Figs. 2E and 2F). *CsGAPDH4* had high transcriptional levels in both callus and flower (Fig. 2H). These results suggest that *CsGAPDH* genes might play various roles at different stages throughout sweet orange development.

### Subcellular localization of CsGAPDH proteins

**Figure 3:**
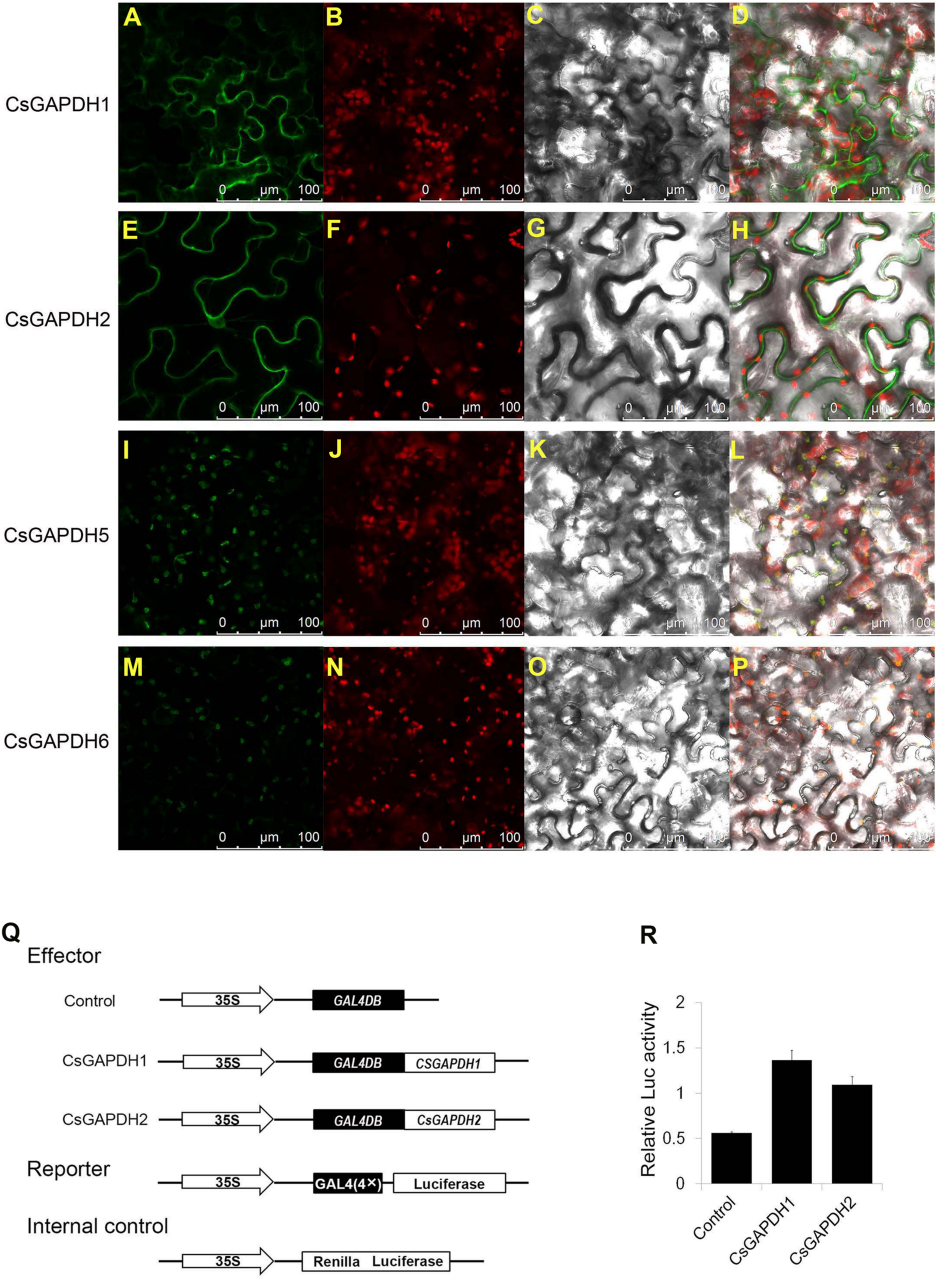
Subcellular localization and transcriptional regulation of CsGAPDHs proteins. (A, E, I, M) Analysis of the subcellular localization of the CsGAPDHs protein in tobacco leaf. The merged pictures (D, H, L, P) include the green fluorescence channel (first panels), the chloroplast autofluorescence channel (second panels) (B, F, J, N) and the bright field channel (third panels) (C, G, K, O). (Q) The main components of the vectors (effector, reporter, and the Internal control) are displayed. (R) Relative luciferase activity of CsGAPDH1 and CsGAPDH2 on promoters of the GAL4 using a dual-luciferase transient assay in *Arabidopsis* protoplasts with GAL4DB as control.

In order to examine CsGAPDHs localization in plant cells, full-length cDNAs of CsGAPDH1, CsGAPDH2, CsGAPDH5 and CsGAPDH6 were fuzed to Green Fluorescent Protein (GFP) driven by the CaMV 35S promoter, which was then transiently expressed through tobacco leaf injections. CsGAPDH1 and CsGAPDH2 were both identified within the nucleus and cytoplasm while CsGAPDH5 and CsGAPDH6 were observed solely in the chloroplast as shown in Figs. 3A–3P. The singular locations of CsGAPDH5 and CsGAPDH6 were consistent with previous findings of leaf-specific expression and group III-specific classification (Figs. 1A, 2I, and 2J).

### Transcriptional activation of CsGAPDH proteins

Recent research has shown that GAPDH proteins within the nucleus have the ability to bind DNA and play a role in transcriptional activation *(Testard et al., 2016*; Zhang, Zhao & Zhou, 2017). To determine the functions of nuclear localized CsGAPDHs in gene expression regulation, CsGAPDH1 and CsGAPDH2 were selected for dual-luciferase transient assays using *Arabdopsis* protoplasts (Figs. 3Q and 3R). CsGAPDH proteins were fuzed with 35S-GAL4DB as effectors, and 4×GAL4 was fuzed to luciferase driven by the CaMV 35S promoter as a reporter. Ranilla luciferase driven by the *Arabidopsis* UBIQUITIN3 promoter was used as an internal control. When CsGAPDH1, CsGAPDH2 and the control vector were transformed into protoplasts, it was discovered that CsGAPDH1 and CsGAPDH2 were capable of enhancing luciferase activity at levels about 2-fold higher than the internal control, indicating that both of these proteins function as transcriptional activators (Figs. 3Q and 3R).

### Light-responsive expression dynamics of *CsGAPDH* genes

**Figure 4:**
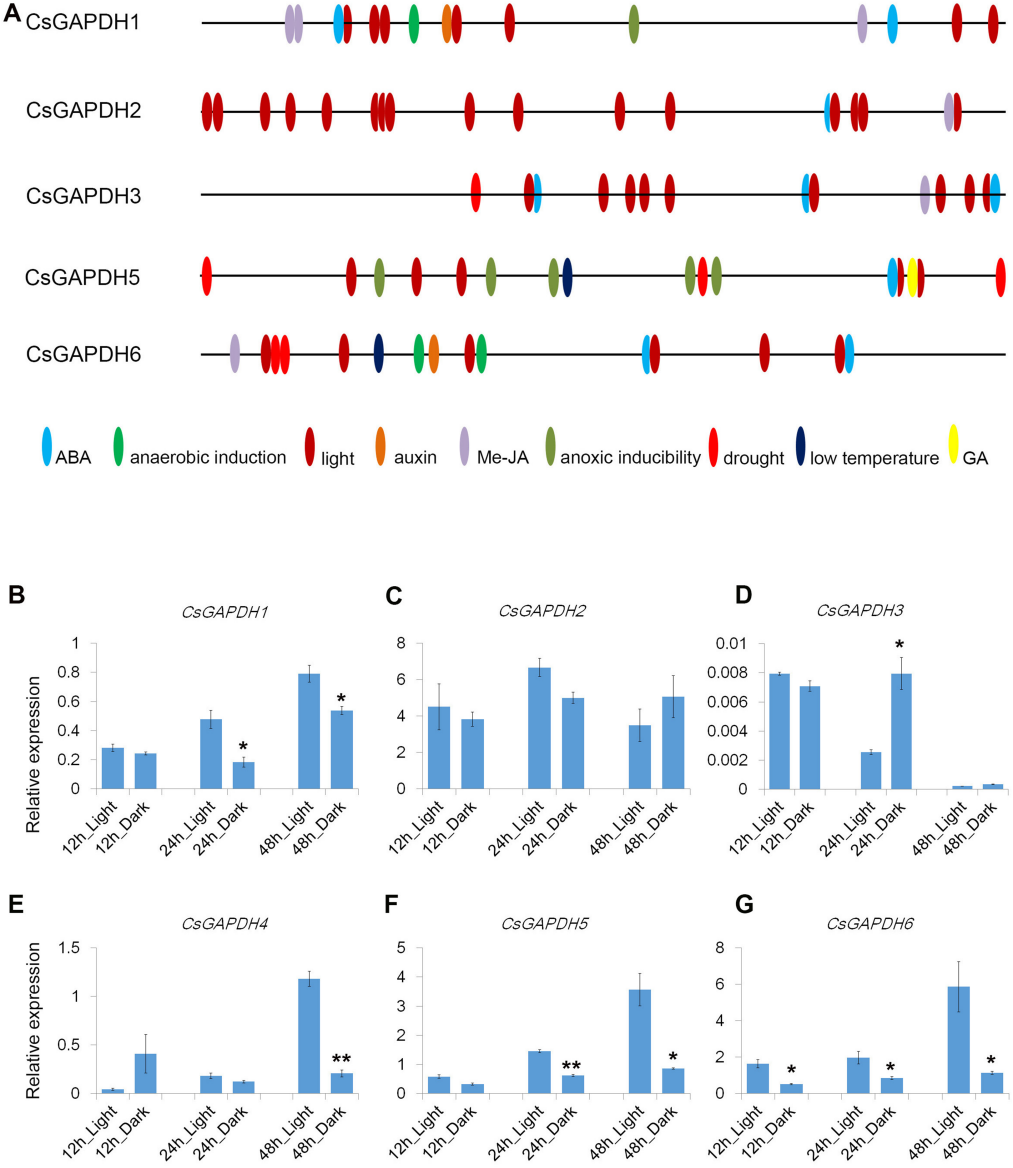
Cis-elements in promoters and expression of *CsGAPDH* genes under darkness treatment. (A) The 1500 bp genomic DNA sequences upstream *CsGAPDH* genes were submitted to PLACEcare web server and then to identify the putative cis-acting regulatory elements. (B–G) Expression level of six *CsGAPDH* genes under dark condition at 12 h, 24 h and 48 h, with the *C. sinensis* actin gene as internal control. Error bars denotes the standard deviation calculated from three independent experiments, statistical significance was analyzed by Student’s *t*-test (**p < 0.01, *p < 0.05).

Cis-elements are important components for protein binding sites on promoters in order to regulate transcription in response to phytohormones and other stress factors. A total of 1,500 bp upstream flanks of *CsGAPDH* genes were analyzed using the online software PlantCARE (http://bioinformatics.psb.ugent.be/webtools/plantcare/html/). Nine types of cis-acting elements were detected in the promoter regions of five *CsGAPDH* genes, including ABA, Me-JA, auxin, and a GA responsive element (Fig. 4A). In addition, stress responsive elements related to low temperature and drought conditions were also been identified. Notably, light-responsive elements were the most abundant elements and were present in multiple locations of each *CsGAPDH* promoter region, indicating that these *CsGAPDH* genes are potentially regulated by light.

Next, the expression dynamics of *CsGAPDH* genes in response to light were examined. Expression of *CsGAPDH* genes in seedlings undergoing dark treatment for 12 h, 24 h, and 48 h were compared with an untreated control. *CsGAPDH1* was down-regulated at 24 h and 48 h during dark treatment (Fig. 4B), whereas *CsGAPDH2* presented a slightly lower expression at 24 h (Fig. 4C). *CsGAPDH3* showed an increased expression level at 24 h (Fig. 4D), while CsGAPDH4 demonstrated this level at only 12 h (Fig. 4E). *CsGAPDH4* was down-regulated in response to darkness treatment at 48 h (Fig. 4E). Subgroup III genes (*CsGAPDH5* and *CsGAPDH6*) showed much lower transcriptional levels than the control at all the time points (Figs. 4F and 4G), which were consistent with their leaf specific expression (Figs. 2I and 2J). These results align with the abundance of light-responsive elements identified, indicating that CsGAPDH proteins may play roles within metabolism pathways related to photosynthesis and other light response pathways.

### Interaction analysis of CsGAPDH protein

**Figure 5:**
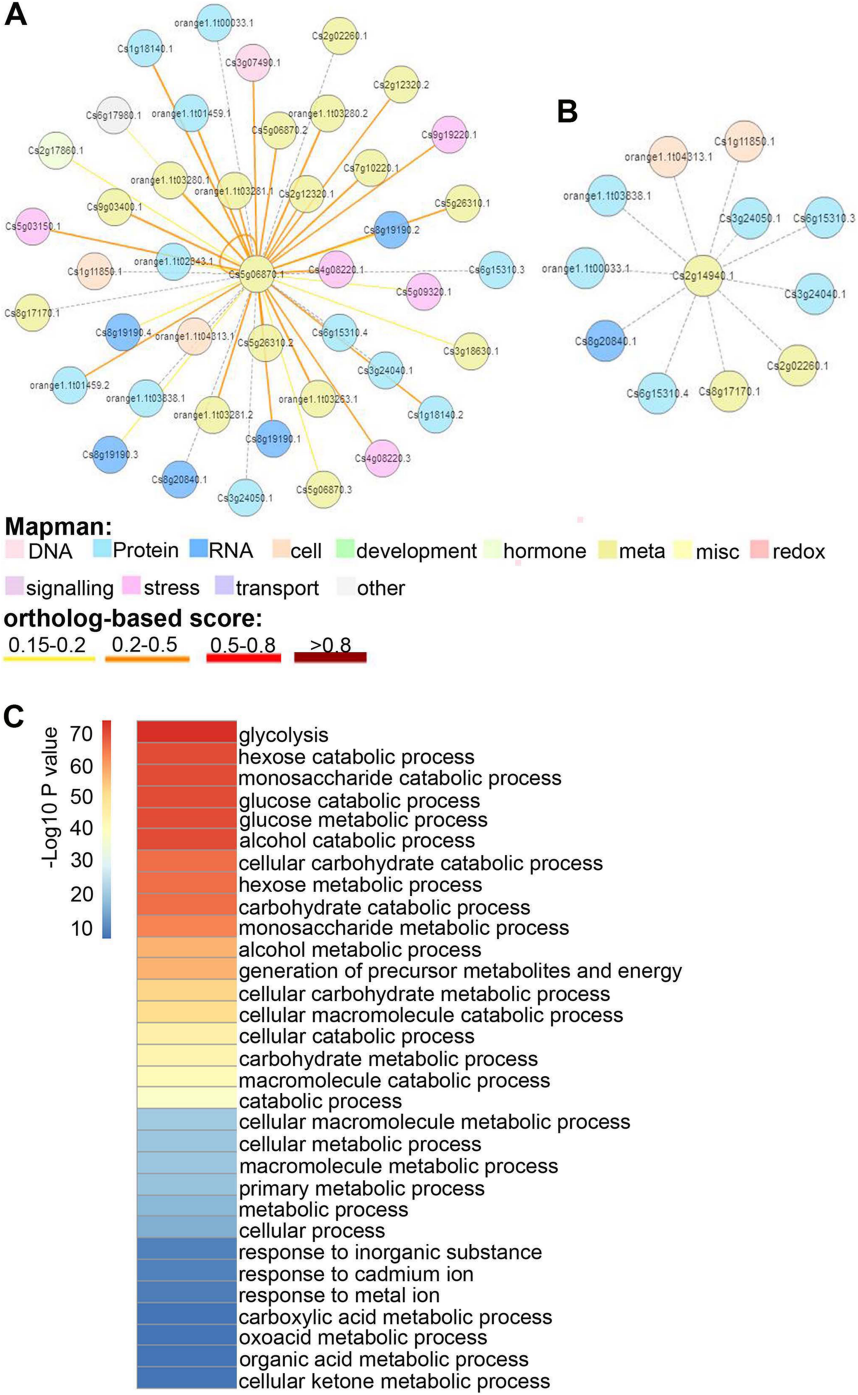
The protein–protein interactions (PPI) of GAPDH proteins in sweet orange. (A–B) Orthologous-based and domain-based methods were employed to predict PPI networks of CsGAPDH2 and CsGAPDH6 in sweet orange. The colors represent different functions of protein in rectangle. (C) Enriched GO analysis of putative CsGAPDHs-interacting proteins.

To discern the functions of CsGAPDHs in sweet orange, protein–protein interaction (PPI) networks of CsGAPDH proteins were built using the *C. sinensis* annotation project database. CsGAPDH1, CsGAPDH2, and CsGAPDH4 shared more than 90% of their interacting proteins, and in turn, all three proteins can interact with each other (Fig. 5A; Figs. S2 and S3). Meanwhile, it is predicted that CsGAPDH1, CsGAPDH2, and CsGAPDH4 can interact with themselves. CsGAPDH3 and CsGAPDH6 were predicted to interact with ten of the same proteins (Fig. 5B; Fig. S4).

To determine the functions of these interacting proteins, GO enrichment analysis for these proteins was performed with the AgriGO tools (http://bioinfo.cau.edu.cn/agriGO/analysis.php), reflecting the possible biological process that they involved in (Fig. 5C). *Arabidopsis* is considered one of the most important model organisms for plants and the majority of its genes have been functionally characterized. This makes it a good starting point to determine the functionality of the proteins found to interact with CsGAPDHs in sweet orange. According to their homologous proteins in *Arabidopsis*, these proteins appear to play roles in multiple biological metabolism processes such as glycolysis, generation of precursor metabolites and energy, carbohydrate catabolic process, glucose catabolic process, and more (Fig. 5C). Moreover, these proteins also are involved in response to inorganic substances such as cadmium ions or metal ions. Through extensive analysis, these findings suggest that CsGAPDHs may interact with each other to form complexes for cooperation or feedback networks.

### Expression profiles of *CsGAPDH* genes in response to phosphorus deficiency in the root of *Poncirus trifoliata*

**Figure 6:**
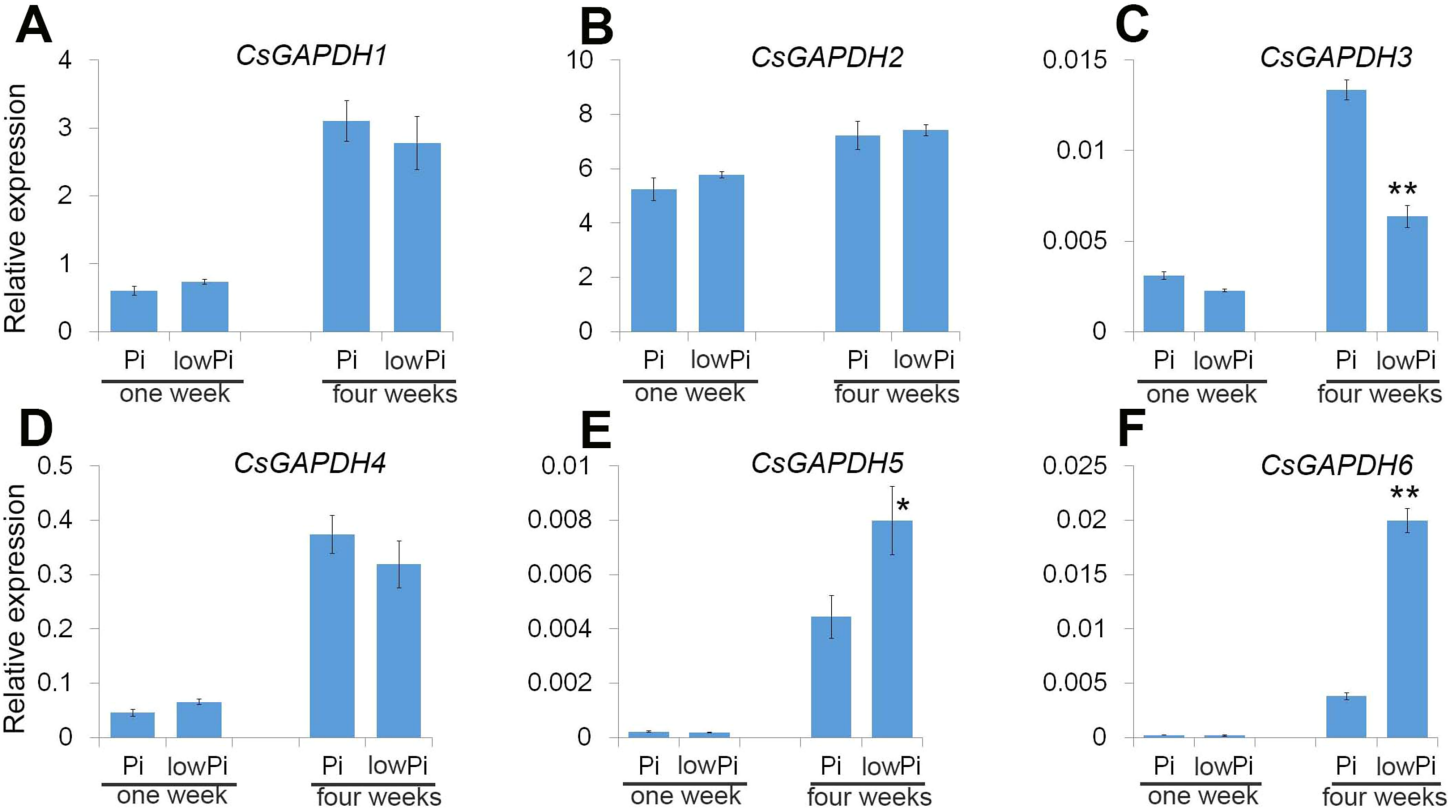
The *CsGAPDH* genes expression under Pi-deficiency in *P. trifoliata*. (A–F) Expression level of six *CsGAPDH* genes under Pi-defificiency treatment at 1 week and 4 weeks, with the Citrus *actin* gene as internal control. Error bars denotes the standard deviation calculated from three independent experiments, statistical significance was analyzed by Student’s *t*-test (**p < 0.01, *p < 0.05).

Sweet orange is generally used as a scion in agricultural production, and *Poncirus trifoliata* (Pt) is the main rootstock for sweet orange due to its ideal growth characteristics such as a strong root system, drought and pest resistance. Phosphorus (Pi) is important macronutrient for plant growth and development, and the *CsGAPDH* genes involved in response to inorganic substance (Fig. 5C). To gain a deeper understanding of *CsGAPDH* genes, the expression of *CsGAPDH* genes in roots exposed to phosphorus deficiency for 1 week and 4 weeks, with the seedling in the normal condition as control (Data S2) were investigated. *CsGAPDH5* and *CsGAPDH6* were predominantly expressed in young leaves, with *CsGAPDH3* showing significantly low expression in all the tissues (Figs. 2G, 2I, and 2J). Consistent with this, *CsGAPDH3*, *CsGAPDH5*, and *CsGAPDH6* showed much lower expression when compared with *CsGAPDH1*, *CsGAPDH2*, and *CsGAPDH4* at the first and fourth week with or without Pi (Figs. 6A–6F). In addition, *CsGAPDH5* and *CsGAPDH6* were relatively highly expressed during the Pi starvation treatment by the fourth week (Figs. 6E and 6F), whereas *CsGAPDH3* showed opposite pattern (Fig. 6C). However, *CsGAPDH1*, *CsGAPDH2* and *CsGAPDH4* showed high transcriptional accumulation with or without Pi starvation treatment (Figs. 6A, 6B, and 6D). In conclusion, the expression of CsGAPDH3, CsGAPDH5, and CsGAPDH6 exhibited a time-dependent response to Pi deficiency in Pt roots.

### Expression of *CsGAPDH* genes during seeds germination

**Figure 7:**
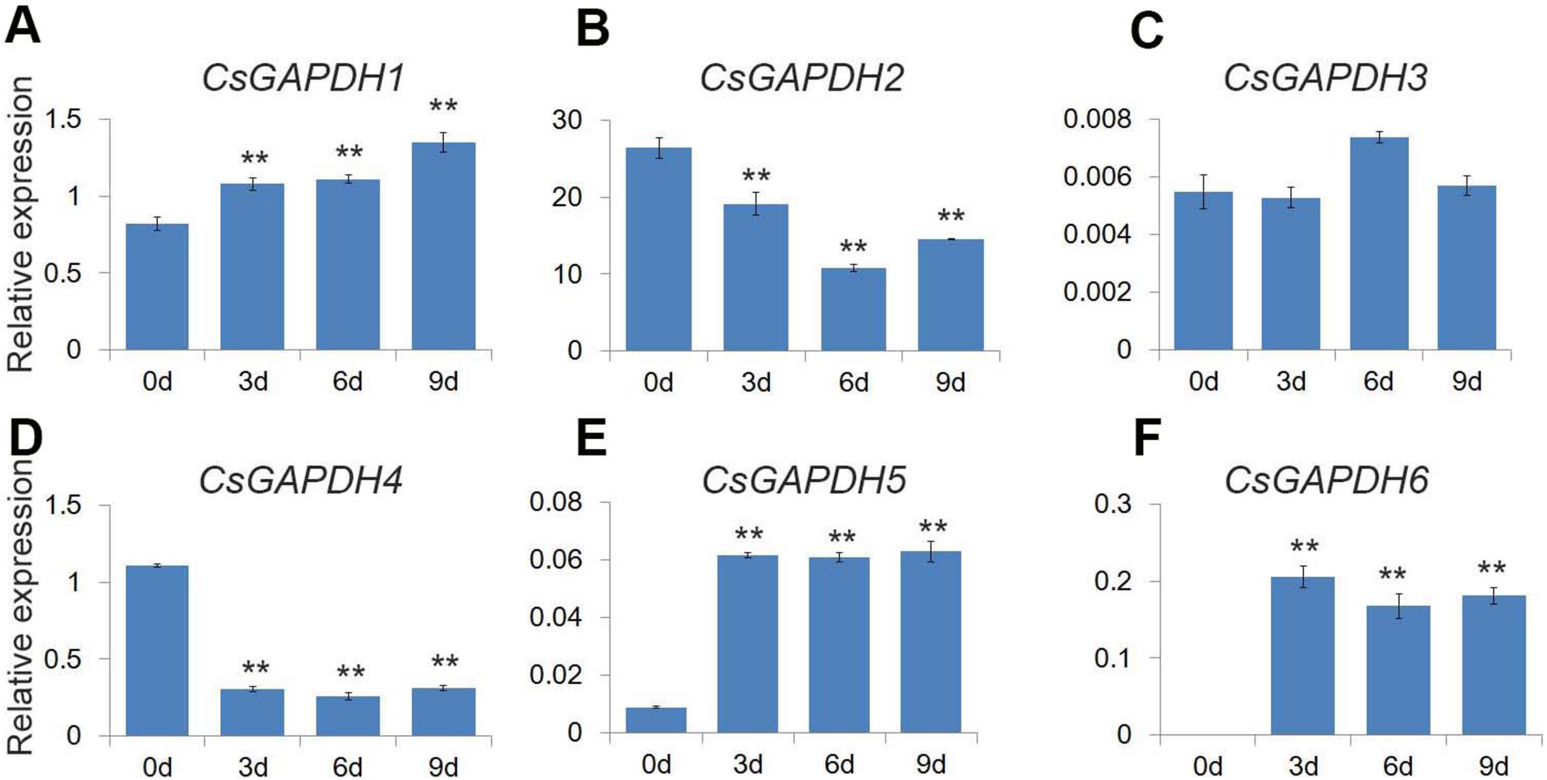
Expression of *CsGAPDH* genes during seed germination of sweet orange. (A–F) Expression of *CsGAPDH* Genes during seed germination of sweet orange. Expression level of six *CsGAPDH* genes at 0 d, 3 d, 6 d and 9 d after seeds germination, with the *C. sinensis actin* gene as internal control. Error bars denotes the standard deviation calculated from three independent experiments, statistical significance was analyzed by Student’s *t*-test (**p < 0.01, *p < 0.05).

GO analysis revealed that the *CsGAPDH* genes may be involved in energy metabolic processes, providing energy for plant growth and development. Previous studies showed that glycolysis and TCA cycle provide considerable energy for germination through increase *GAPDHs* levels (Kim et al., 2009). In addition, plastidial glyceraldehyde-3-phosphate dehydrogenases are involved in ABA signal transduction, affecting seed germination (Muñoz-Bertomeu et al., 2011). Citrus seeds are relatively sensitive to salt stress during germination (Ziogas et al., 2017). The expression levels of *CsGAPDHs* during germination were monitored to uncover additional functions of the *CsGAPDH* genes. The expression of *CsGAPDH1*, *CsGAPDH5* and *CsGAPDH6* were increased at the third, sixth, and ninth day (Figs. 7A, 7E, and 7F). On the other hand, the expression of *CsGAPDH2* and *CsGAPDH4* decreased significantly subsequent to 3 days after germination (Figs. 7B and 7D). Therefore, it is speculated that some *CsGAPDHs* may be involved in energy metabolism during seed germination.

## Discussion

In tobacco, NtGAPC1 and NtGAPC2 demonstrated nucleo-cytosolic localization and were able to bind nucleic acids (Testard et al., 2016). Rice OsGAPDH1 was located in both the cytoplasm and nucleus, and was shown to be involved in transcriptional regulation (Zhang, Zhao & Zhou, 2017). Both CsGAPDH1 and CsGAPDH2 were detected in the cytoplasm and nucleus, with transcriptional activation in protoplasts (Figs. 3A–3H). It can be inferred that CsGAPDH1 and CsGAPDH2 may shuttle between the cytoplasm, nucleus and other organelles, and regulate the expression of glycolysis and stress-related genes like that in rice and *Arabidopsis* (Guo et al., 2012; Zhang, Zhao & Zhou, 2017). Although CsGAPDH1 was not detected in the chloroplast, *CsGAPDH1* was down-regulated at the 24 h and 48 h time points under dark treatment (Fig. 4B). Consistent with these results, light-responsive elements were identified in several regions of *CsGAPDH1* promoter (Fig. 4A).

GAPDH serves as a key regulator in promoting seedling growth under low levels of selenium in *Arabidopsis* (Takeda & Fukui, 2015). *AtGAPC1* expression and nuclear accumulation were induced under Cadmium treatment (Vescovi et al., 2013). The results of GO analysis for all the genes interacting with CsGAPDHs indicated that CsGAPDHs may participate in response to inorganic substances (Fig. 5C). Under phosphorus deficiency conditions, *CsGAPDH3* and *CsGAPDH*6 were down-regulated and up-regulated respectively (Figs. 6C and 6F), indicating GAPDHs function in some processes related to phosphorus.

There are many genome functional annotation methods available to annotate genomes with new tools being developed every year. Multiple methods were used in this study to evaluate and compare genome annotations, each having its own advantages and disadvantages (Bakke et al., 2009; Kalkatawi, Alam & Bajic, 2015; Kasukawa et al., 2003, Liu, Ma & Goryanin, 2013; Yang, Gilbert & Kim, 2010). It is not reasonable to expect all the annotation results from one method to be correct. For example, in order to identity the CsGAPDH proteins in citrus, word “Glyceraldehyde-3” was searched for in the “C.sinensis_v2.0_HZAU_csi.gene.models.gff3” file which was downloaded from ftp://ftp.bioinfo.wsu.edu/www.citrusgenomedb.org/Citrus_sinensis/C.sinensis_Hzau_v2.0_genome/annotation/, and 17 candidates were identified. From there, eight putative *CsGAPDH* genes were identified by removal of different transcripts in the same gene. Moreover, to search for the presence of conserved Gp_dh_N or Gp_dh_C domains, the candidates were examined using the online program SMART (http://smart.embl-heidelberg.de/). Finally, six candidates were confirmed to be *CsGAPDH* by the presence of Gp_dh_N and/or Gp_dh_C domains. The other two candidates were identified as containing an Aldedh domain but not Gp_dh_N or Gp_dh_C domains, suggesting that they may be acetaldehyde dehydrogenase related proteins.

Together with existing research, this study shows that GAPDHs play various roles in addition to glycolysis, such as in response to light, phosphorus deficiency, and transcriptional regulation, which may be helpful for future exploration of the biological functions of *CsGAPDH* genes.

## Conclusions

In conclusion, this paper presents genome-wide analysis of six *CsGAPDH* genes in the sweet orange genome. These genes have different expression patterns in different tissues and during the seed germination process, with expression levels of some *CsGAPDH* genes influenced by light and phosphorus deficiency. Moreover, subcellular localization and cis-elements of the promoters were demonstrated. Predictions for novel genes that interact with the CsGAPDH family are presented in this study. These findings may be helpful for future exploration of the biological functions of *CsGAPDH* genes.

## Supplemental Information

10.7717/peerj.7934/supp-1 Supplemental Information 1 Sequence alignments of GAPDH sequences of sweet orange from two databases (http://citrus.hzau.edu.cn/orange/ and https://phytozome.jgi.doe.gov/pz/portal.html#!info?alias=Org_Csinensis).

10.7717/peerj.7934/supp-2 Supplemental Information 2 Orthologous-based and domain-based methods were employed to predict PPI network of CsGAPDH1 in sweet orange.

10.7717/peerj.7934/supp-3 Supplemental Information 3 Orthologous-based and domain-based methods were employed to predict PPI network of CsGAPDH4 in sweet orange.

10.7717/peerj.7934/supp-4 Supplemental Information 4 Orthologous-based and domain-based methods were employed to predict PPI network of CsGAPDH3 in sweet orange.

10.7717/peerj.7934/supp-5 Supplemental Information 5 GAPDH sequence of six citrus species. The meaning of the letters in front of the gene Locus is as follows: Cs (sweet orange, *C. sinensis*); Ciclev (clementine mandarine, *Citrus reticulata*); Cg (pummelo, *Citrus grandis*); Ci (papeda, *Citrus ichangenis*); Cm (citron, *Citrus medica*)；sb (Chinese box orange, *Atalantia buxifolia*).

10.7717/peerj.7934/supp-6 Supplemental Information 6 Relative expression of *CsGAPDH* genes after phosphorus deficiency treatment.

10.7717/peerj.7934/supp-7 Supplemental Information 7 Basic information of *CsGAPDH* family.

10.7717/peerj.7934/supp-8 Supplemental Information 8 List of primer sequences.

## Additional Information and Declarations

### Data Availability

The following information was supplied regarding data availability: The raw measurements, GAPDH gene accession numbers, amino acid sequences for multiple alignments and RT-PCR results are available in the Supplemental Files.
