# Supplementary figures and images for: Genome-wide identification, characterization, interaction network and expression profile of GAPDH gene family in sweet orange (Citrus sinensis)
Source: PeerJ. 2019 Nov 14;7:e7934. doi: 10.7717/peerj.7934 (PMC6858985; doi:10.7717/peerj.7934)

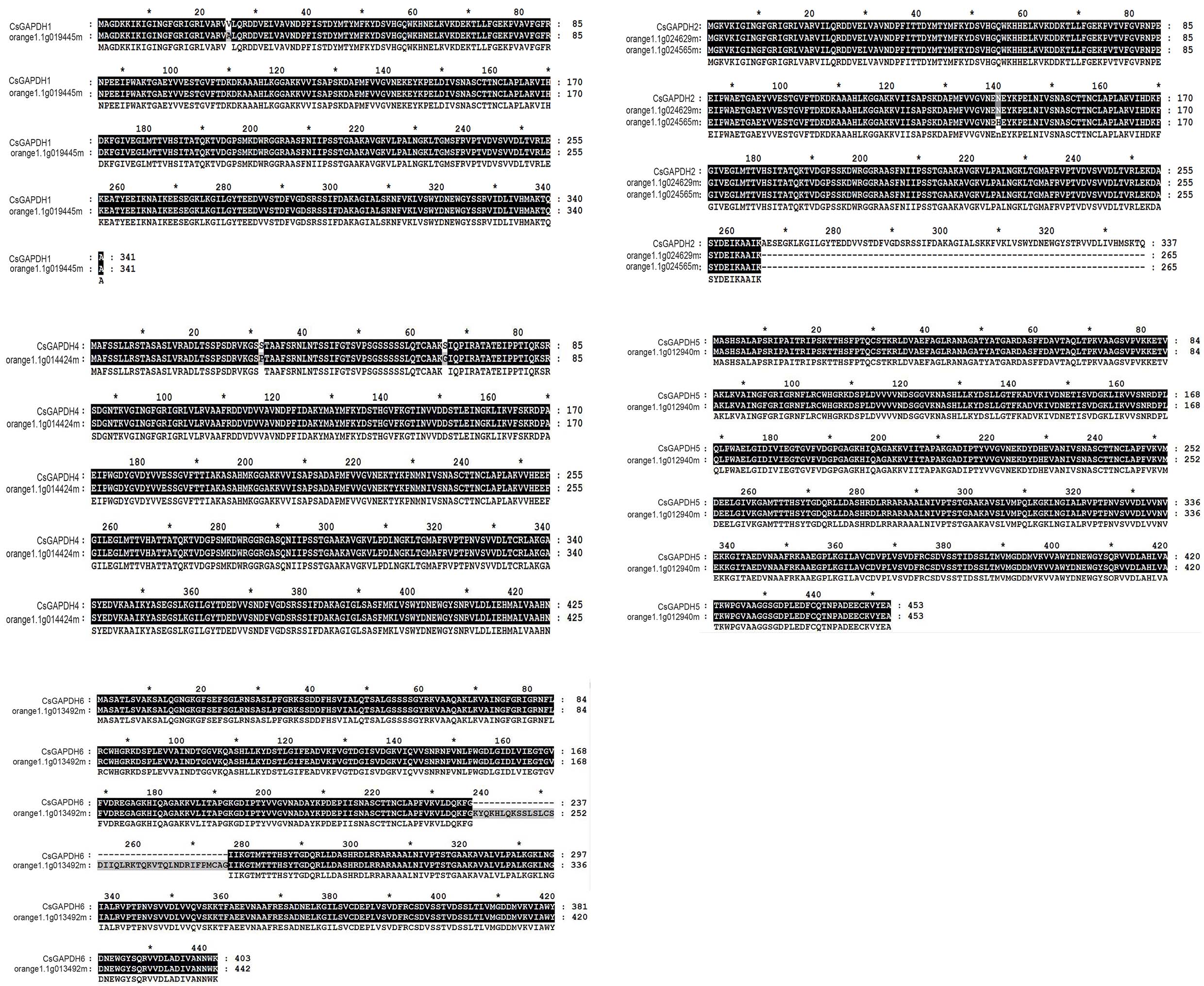

Supplement: Supplemental Information 1 [file peerj-07-7934-s001.jpg]

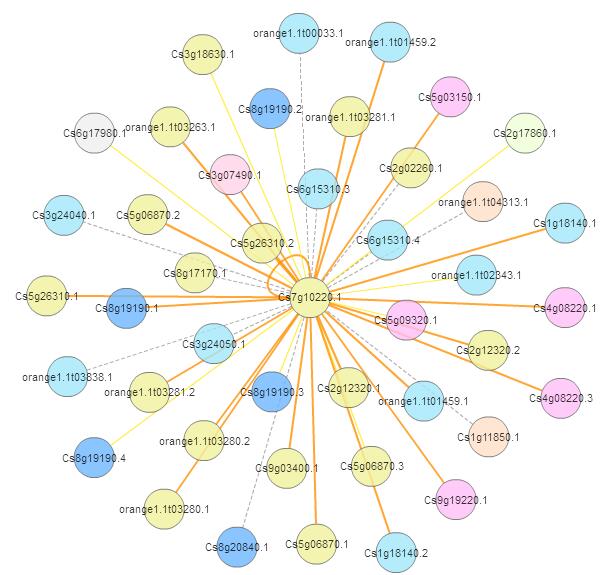

Supplement: Supplemental Information 2 [file peerj-07-7934-s002.jpg]

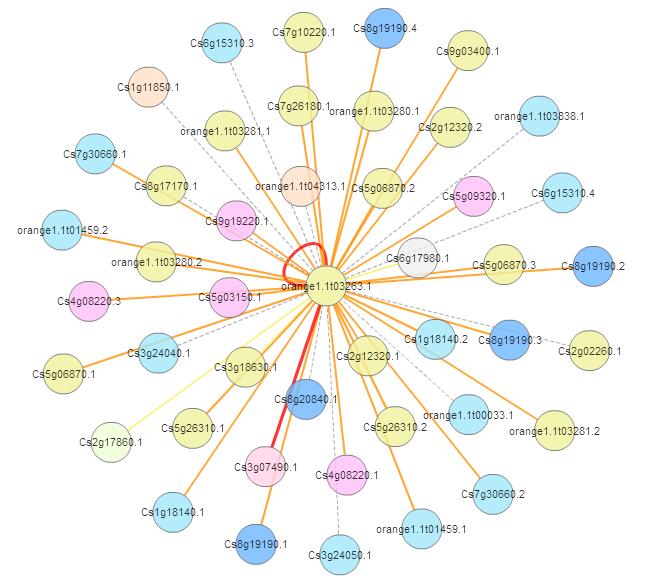

Supplement: Supplemental Information 3 [file peerj-07-7934-s003.jpg]

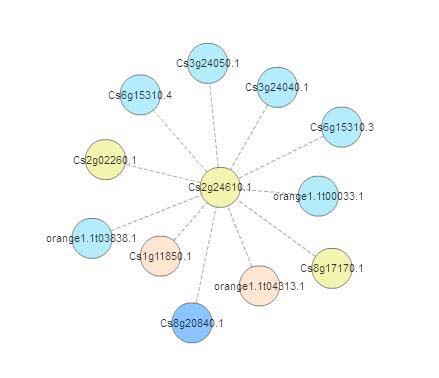

Supplement: Supplemental Information 4 [file peerj-07-7934-s004.jpg]
