# Supplementary material for: Genome-wide identification, characterization, interaction network and expression profile of GAPDH gene family in sweet orange (Citrus sinensis)
Source: PeerJ. 2019 Nov 14;7:e7934. doi: 10.7717/peerj.7934 (PMC6858985; doi:10.7717/peerj.7934)
Supplement: Supplemental Information 5 — The meaning of the letters in front of the gene Locus is as follows: Cs (sweet orange, C. sinensis); Ciclev (clementine mandarine, Citrus reticulata); Cg (pummelo, Citrus grandis); Ci (papeda, Citrus ichangenis); Cm (citron, Citrus medica)；sb (Chinese box orange, Atalantia buxifolia). [file peerj-07-7934-s005.doc]

>CsGAPDH1

MAGDKKIKIGINGFGRIGRLVARVVLQRDDVELVAVNDPFISTDYMTYMFKYDSVHGQWKHNELKVKDEKTLLFGEKPVAVFGFRNPEEIPWAKTGAEYVVESTGVFTDKDKAAAHLKGGAKKVVISAPSKDAPMFVVGVNEKEYKPELDIVSNASCTTNCLAPLAKVIHDKFGIVEGLMTTVHSITATQKTVDGPSMKDWRGGRAASFNIIPSSTGAAKAVGKVLPALNGKLTGMSFRVPTVDVSVVDLTVRLEKEATYEEIKNAIKEESEGKLKGILGYTEEDVVSTDFVGDSRSSIFDAKAGIALSKNFVKLVSWYDNEWGYSSRVIDLIVHMAKTQA

>CsGAPDH2

MGKVKIGINGFGRIGRLVARVILQRDDVELVAVNDPFITTDYMTYMFKYDSVHGQWKHHELKVKDDKTLLFGEKPVTVFGVRNPEEIPWAETGAEYVVESTGVFTDKDKAAAHLKGGAKKVIISAPSKDAPMFVVGVNENEYKPELNIVSNASCTTNCLAPLAKVIHDKFGIVEGLMTTVHSITATQKTVDGPSSKDWRGGRAASFNIIPSSTGAAKAVGKVLPALNGKLTGMAFRVPTVDVSVVDLTVRLEKDASYDEIKAAIKAESEGKLKGILGYTEDDVVSTDFVGDSRSSIFDAKAGIALSKKFVKLVSWYDNEWGYSTRVVDLIVHMSKTQ

>CsGAPDH3

MMTRNPEEIPWAETGAELAVESTGVFTDKDKAAAHLKGGTKKVVISAPSKDAPMFVMGVNEKEYKKELDIVSNASCTTTCLASLAKVIQNKFGIVEGFMTTVHAIAATQKTVDGPSMKDWRRGRAASFNIIPSSDGAAKEYMVDLTVRLEKKATYDDIKAALKEASQGEMKGIFGYIEDDVVSTDFVGDNRSSIFDAKAGISLSDNLEKLVSWYDTDAAFIFWIGVGILRLRVWSGSGSRLFT

>CsGAPDH4

MAFSSLLRSTASASLVRADLTSSPSDRVKGSSTAAFSRNLNTSSIFGTSVPSGSSSSSLQTCAAKSIQPIRATATEIPPTIQKSRSDGNTKVGINGFGRIGRLVLRVAAFRDDVDVVAVNDPFIDAKYMAYMFKYDSTHGVFKGTINVVDDSTLEINGKLIKVFSKRDPAEIPWGDYGVDYVVESSGVFTTIAKASAHMKGGAKKVVISAPSADAPMFVVGVNEKTYKPNMNIVSNASCTTNCLAPLAKVVHEEFGILEGLMTTVHATTATQKTVDGPSMKDWRGGRGASQNIIPSSTGAAKAVGKVLPDLNGKLTGMAFRVPTPNVSVVDLTCRLAKGASYEDVKAAIKYASEGSLKGILGYTDEDVVSNDFVGDSRSSIFDAKAGIGLSASFMKLVSWYDNEWGYSNRVLDLIEHMALVAAHN

>CsGAPDH5

MASHSALAPSRIPAITRIPSKTTHSFPTQCSTKRLDVAEFAGLRANAGATYATGARDASFFDAVTAQLTPKVAAGSVPVKKETVAKLKVAINGFGRIGRNFLRCWHGRKDSPLDVVVVNDSGGVKNASHLLKYDSLLGTFKADVKIVDNETISVDGKLIKVVSNRDPLQLPWAELGIDIVIEGTGVFVDGPGAGKHIQAGAKKVIITAPAKGADIPTYVVGVNEKDYDHEVANIVSNASCTTNCLAPFVKVMDEELGIVKGAMTTTHSYTGDQRLLDASHRDLRRARAAALNIVPTSTGAAKAVSLVMPQLKGKLNGIALRVPTPNVSVVDLVVNVEKKGITAEDVNAAFRKAAEGPLKGILAVCDVPLVSVDFRCSDVSSTIDSSLTMVMGDDMVKVVAWYDNEWGYSQRVVDLAHLVATKWPGVAAGGSGDPLEDFCQTNPADEECKVYEA

>CsGAPDH6

MASATLSVAKSALQGNGKGFSEFSGLRNSASLPFGRKSSDDFHSVIALQTSALGSSSSGYRKVAAQAKLKVAINGFGRIGRNFLRCWHGRKDSPLEVVAINDTGGVKQASHLLKYDSTLGIFEADVKPVGTDGISVDGKVIQVVSNRNPVNLPWGDLGIDLVIEGTGVFVDREGAGKHIQAGAKKVLITAPGKGDIPTYVVGVNADAYKPDEPIISNASCTTNCLAPFVKVLDQKFGIIKGTMTTTHSYTGDQRLLDASHRDLRRARAAALNIVPTSTGAAKAVALVLPALKGKLNGIALRVPTPNVSVVDLVVQVSKKTFAEEVNAAFRESADNELKGILSVCDEPLVSVDFRCSDVSSTVDSSLTLVMGDDMVKVIAWYDNEWGYSQRVVDLADIVANNWK

>Ciclev10005029m

MAFSSLLRSTASASLVRADLTSSPSDRVKGSSTAAFSRNLNTSSIFGTSVPSGSSSSSLQTCAAKSIQPIRATATEIPPTIQKSRSDGNTKVGINGFGRIGRLVLRVAAFRDDVDVVAVNDPFIDAKYMAYMFKYDSTHGVFKGTINVVDDSTLEINGKLIKVFSKRDPAEIPWGDYGVDYVVESSGVFTTIAKASAHMKGGAKKVVISAPSADAPMFVVGVNEKTYKPNMNIVSNASCTTNCLAPLAKVVHEEFGILEGLMTTVHATTATQKTVDGPSMKDWRGGRGASQNIIPSSTGAAKAVGKVLPDLNGKLTGMAFRVPTPNVSVVDLTCRLAKGASYEDVKAAIKYASEGSLKGILGYTDEDVVSNDFVGDSRSSIFDAKAGIGLSASFMKLVSWYDNEWGYSNRVLDLIEHMALVAAHN

>Ciclev10021037m

MGKVKIGINGFGRIGRLVARVILQRDDVELVAVNDPFITTDYMTYMFKYDSVHGQWKHHELKVKDDKTLLFGEKPVTVFGVRNPEEIPWAETGAEYVVESTGVFTDKDKAAAHLKGGAKKVIISAPSKDAPMFVVGVNEHEYKPELNIVSNASCTTNCLAPLAKVIHDKFGIVEGLMTTVHSITATQKTVDGPSSKDWRGGRAASFNIIPSSTGAAKAVGKVLPALNGKLTGMAFRVPTVDVSVVDLTVRLEKDASYDEIKAAIKAESEGKLKGILGYTEDDVVSTDFVGDSRSSIFDAKAGIALSKKFVKLVSWYDNEWGYSTRVVDLIVHMAKTQ

>Ciclev10032014m

MAGDKKIKIGINGFGRIGRLVARVALQRDDVELVAVNDPFISTDYMTYMFKYDSVHGQWKHNELKVKDEKTLLFGEKPVAVFGFRNPEEIPWAKTGAEYVVESTGVFTDKDKAAAHLKGGAKKVVISAPSKDAPMFVVGVNEKEYKPELDIVSNASCTTNCLAPLAKVIHDKFGIVEGLMTTVHSITATQKTVDGPSMKDWRGGRAASFNIIPSSTGAAKAVGKVLPALNGKLTGMSFRVPTVDVSVVDLTVRLEKEATYEEIKNAIKEESEGKLKGILGYTEEDVVSTDFVGDSRSSIFDAKAGIALSKNFVKLVSWYDNEWGYSSRVIDLIVHMAKTQA

>Ciclev10001114m

MASHSALAPSRIPAITRIPSKTTHSFPTQCSTKRLDVAEFAGLRANAGATYATGARDASFFDAVTAQLTPKVAAGSVPVKKETVAKLKVAINGFGRIGRNFLRCWHGRKDSPLDVVVVNDSGGVKNASHLLKYDSLLGTFKADVKIVDNETISVDGKLIKVVSNRDPLQLPWAELGIDIVIEGTGVFVDGPGAGKHIQAGAKKVIITAPAKGADIPTYVVGVNEKDYDHEVANIVSNASCTTNCLAPFVKVMDEELGIVKGAITTTHSYTGDQRLLDASHRDLRRARAAALNIVPTSTGAAKAVSLVLPQLKGKLNGIALRVPTPNVSVVDLVVNVEKKGITAEDVNAAFRKAAEGPLKGILAVCDVPLVSVDFRCSDVSSTIDSSLTMVMGDDMVKVVAWYDNEWGYSQRVVDLAHLVATKWPGVAAGGSGDPLEDFCQTNPADEECKVYEA

>Ciclev10015476m

MASATLSVAKSTLQGNGKGFSEFSGLRNSASLPFGRKSSDDFHSVIALQTSALGSSSSGYRKVAAQAKLKVAINGFGRIGRNFLRCWHGRKDSPLEVVAINDTGGVKQASHLLKYDSTLGIFEADVKPVGTDGISVDGKVIQVVSNRNPVNLPWGDLGIDLVIEGTGVFVDREGAGKHIQAGAKKVLITAPGKGDIPTYVVGVNADAYKPDEPIISNASCTTNCLAPFVKVLDQKFGIIKGTMTTTHSYTGDQRLLDASHRDLRRARAAALNIVPTSTGAAKAVALVLPALKGKLNGIALRVPTPNVSVVDLVVQVSKKTFAEEVNAAFRESADNELKGILSVCDEPLVSVDFRCSDVSSTVDSSLTLVMGDDMVKVIAWYDNEWGYSQRVVDLADIVANNWK

>Ciclev10017767m

SIFSQHKDSKTLLFGEKPDAVFCVRFLYKFFVPYMNPEEIPWAETGAELAVESTGVFTDKDKAAAHLKGGTKKVVISAPSKDAPMFVMGVNEKEYKKELDIVSNASCTTTCLASLAKVIQNKFGIVEGFMTTVHAIAATQKTVDGPSMKDWRRGRAASFNIIPSSDGAAKEYMVDLTVRLEKKATYDDIKAALKEASQGEMKGIFGYIEDDVVSTDFVGDNRSSIFDAKAGISLSDNLEKLVSWYDTDAAFIFWIGVGILRLRVWSGSGSRLFT

>Cg9g029280

MAFSSLLRSTASASLVRADLTSSPSDRVKGSSTVAFSRNLNTSSIFGTSVPSGSSSSSLQTCATKSIQPIRATATEIPPTIQKPRSDGNTKVGINGFGRIGRLVLRVAAFRDDVDVVAVNDPFIDAKYMAYMFKYDSTHGVFKGTINVVDGSTLEINGKLIKVFSKRDPAEIPWGDYGVDYVVESSGVFTTIAKASAHMKGGAKKVVISAPSADAPMFVVGVNEKTYKPNMNIVSNASCTTNCLAPLAKVVHEEFGILEGLMTTVHATTATQKTVDGPSMKDWRGGRGASQNIIPSSTGAAKAVGKVLPDLNGKLTGMAFRVPTPNVSVVDLTCRLAKSASYEDVKAAIKYASEGSLKGILGYTDEDVVSNDFVGDSRSSIFDAKAGIGLSASFMKLVSWYDNEWGYSNRVLDLIEHMALVAARN

>Cg5g006480

MGKVKIGINGFGRIGRLVARVILQRDDVELVAVNDPFITTDYMTYMFKYDSVHGQWKHHELKVKDDKTLLFGEKPVTVFGVRNPEEIPWAETGAEYVVESTGVFTDKDKAAAHLKGGAKKVIISAPSKDAPMFVVGVNENEYKPELNIVSNASCTTNCLAPLAKVIHDKFGIVEGLMTTVHSITATQKTVDGPSSKDWRGGRAASFNIIPSSTGAAKAVGKVLPALNGKLTGMAFRVPTVDVSVVDLTVRLEKDASYDEIKAAIKAESEGKLKGILGYTEDDVVSTDFVGDSRSSIFDAKAGIALSKKFVKLVSWYDNEWGYSTRVVDLIVHMSKTQGSEFK

>Cg7g017610

MAGDKKIKIGINGFGRIGRLVARVALQRDDVELVAVNDPFISTDYMTYMFKYDSVHGQWKHNELKVKDEKTLLFGEKPVAVFGFRNPEEIPWAKTGAEYVVESTGVFTDKDKAAAHLKGGAKKVVISAPSKDAPMFVVGVNEKEYKPELDIVSNASCTTNCLAPLAKVIHDKFGIVEGLMTTVHSITATQKTVDGPSMKDWRGGRAASFNIIPSSTGAAKAVGKVLPALNGKLTGMSFRVPTVDVSVVDLTVRLEKETTYEEIKNAIKEESEGKLKGILGYTEEDVVSTDFVGDSRSSIFDAKAGIALSKNFVKLVSWYDNEWGYSSRVIDLIVHMAKTQA

>Cg3g025510

MASHSALAPSRIPAITRIPSKTTHSFPTQCSTKRLDVAEFAGLRANAGATYATGARDASFFDAVTAQLTPKVASGSVPVKKETMAKLKVAINGFGRIGRNFLRCWHGRKDSPLDVVVVNDSGGVKNASHLLKYDSLLGTFKADVKIVDNETISVDGKLIKVVSNRDPLQLPWAELGIDIVIEGTGVFVDGPGAGKHIQAGAKKVIITAPAKGADIPTYVVGVNEKDYDHEVANIVSNASCTTNCLAPFVKVMDEELGIVKGTMTTTHSYTGDQRLLDASHRDLRRARAAALSIVPTSTGAAKAVSLVLPQLKGKLNGIALRVPTPNVSVVDLVVNVEKKGITAEDVNAAFRKAAEGPLKGILAVCDVPLVSVDFRCSDVSSTIDSSLTMVMGDDMVKVVAWYDNEWGYSQRVVDLAHLVATKWPGVAAGGSGDPLEDFCQTNPADEECKVYEA

>Cg2g009300

MASATLSVAKSALQGNGKGFSEFSGLRNSASLPFGRKSSDDFHSVIALQTSALGSSSSGYRKVAAQAKLKVAINGFGRIGRNFLRCWHGRKDSPLEVVAINDTGGVKQASHLLKYDSTLGIFEADVKPVGTDGISVDGKVIQVVSNRNPVNLPWGDLGIDLVIEGTGVFVDREGAGKHIQAGAKKVLITAPGKGDIPTYVVGVNADAYKPDEPIISNASCTTNCLAPFVKVLDQKFGIIKGTMTTTHSYTGDQRLLDASHRDLRRARAAALNIVPTSTGAAKAVALVLPALKGKLNGIALRVPTPNVSVVDLVVQVSKKTFAEEVNAAFRESADNELKGILSVCDEPLVSVDFRCSDVSSTVDSSLTLVMGDDMVKVIAWYDNEWGYSQRVVDLADIVANNW

> Cg2g011630

MFVMGVNEKEYKKELDIVSNASCTTTCLASLAKVIQNKFGIVEGFMTTVHAIAATQKTVDGPSMKDWRGGRAASFNIIPSSDGAAKEYMVDLTVRLEKKATYDDIKAAHKEASQGEMKGIFGYTEDDVVSTDFVGDNRSSIFYAEAGISLSDNFVKLVSWYDTEWSYSPGGIDLICHIASCR

>Cm303240

MAFSSLLRSTASASLVRADLTSSPSDRVKGSSTAAFSRNLNTSSIFGTSVPSGSSSSSLQTCAAKSIQPIRATATEIPPTIQKPRSDGNTKVGINGFGRIGRLVLRVAAFRDDVDVVAVNDPFIDAKYMAYMFKYDSTHGVFKGTINVVDDSTLEINGKLIKVFSKRDPAEIPWGDYGVDYVVESSGVFTTIAKASAHMKGGAKKVVISAPSADAPMFVVGVNEKTYKPNMNIVSNASCTTNCLAPLAKVVHEEFGILEGLMTTVHATTATQKTVDGPSMKDWRGGRGASQNIIPSSTGAAKAVGKVLPDLNGKLTGMAFRVPTANVSVVDLTCRLAKSASYEDVKAAIKYASEGSLKGILGYTDEDVVSNDFVGDSRSSIFDAKAGIGLSASFMKLVSWYDNEWGYSNRVLDLIEHMALVAARN

>Cm020370

MGKVKIGINGFGRIGRLVARVILQRDDVELVAVNDPFITTDYMTYMFKYDSVHGQWKHHELKVKDDKTLLFGEKPVTVFGVRNPEEIPWAETGAEYVVESTGVFTDKDKAAAHLKGGAKKVIISAPSKDAPMFVVGVNENEYKPELNIVSNASCTTNCLAPLAKVIHDKFGIVEGLMTTVHSITATQKTVDGPSSKDWRGGRAASFNIIPSSTGAAKAVGKVLPALNGKLTGMAFRVPTVDVSVVDLTVRLEKDASYDEIKAAIKAESEGKLKGILGYTEDDVVSTDFVGDSRSSIFDAKAGIALSKKFVKLVSWYDNEWGYSTRVVDLIVHMSKTQ

>Cm302760

MASHSALAPSRIPAITRIPSKTTHSFPTQCSTKRLDVAEFAGLRANAGVTYATGARDASFFEAVTAQLTPKVASGSVPVKKETVAKLKVAINGFGRIGRNFLRCWHGRKDSPLDVVVVNDSGGVKNASHLLKYDSLLGTFKADVKIVDNETISVDGKLIKVVSNRDPLQLPWAELGIDIVIEGTGVFVDGPGAGKHIQAGAKKVIITAPAKGADIPTYVVGVNEKDYDHEVANIVSNASCTTNCLAPFVKVMDEELGIVKGTMTTTHSYTGDQRLLDASHRDLRRARAAALNIVPTSTGAAKAVSLVLPQLKGKLNGIALRVPTPNVSVVDLVVNVEKKGITAEDVNAAFRKAAEGPLKG

ILAVCDVPLVSVDFRCSDVSSTIDSSLTMVMGDDMVKVVAWYDNEWGYR

>Cm122470

MASHSALAPSRIPAITRIPSKTTHSFPTQCSTKRLDVAEFAGLRANAGVTYATGARDASFFEAVTAQLTPKVASGSVPVKKETVAKLKVAINGFGRIGRNFLRCWHGRKDSPLDVVVVNDSGGVKNASHLLKYDSLLGTFKADVKIVDNETISVDGKLIKVVSNRDPLQLPWAELGIDIVIEGTGVFVDGPGAGKHIQAGAKKVIITAPAKGADIPTYVVGVNEKDYDHEVANIVSNASCTTNCLAPFVKVMDEELGIVKGTMTTTHSYTGDQRLLDASHRDLRRARAAALNIVPTSTGAAKAVSLVLPQLKGKLNGIALRVPTPNVSVVDLVVNVEKKGITAEDVNAAFRKAAEGPLKGILAVCDVPLVSVDFRCSDVSSTIDSSLTMVMGDDMVKVVAWYDNEWGYSQRVVDLAHLVATKWPGVAAGGSGDPLEDYCQTNPADEECKVYEA

>Cm129360

MASATLSVAKSALQGNGKGFSEFSGLRNSASLPFGRKSSDDFHSVIALQTSALGSSSSGYRKVAAQAKLKVAINGFGRIGRNFLRCWHGRKDSPLEVVAINDTGGVKQASHLLKYDSTLGIFEADVKPVGTDGISVDGKVIQVVSNRNPVNLPWGDLGIDLVIEGTGVFVDREGAGKHIQAGAKKVLITAPGKGDIPTYVVGVNADAYKPDEPIISNASCTTNCLAPFVKVLDQKFGIIKGTMTTTHSYTGDQRLLDASHRDLRRARAAALNIVPTSTGAAKAVALVLPALKGKLNGIALRVPTPNVSVVDLVVQVSKKTFAEEVNAAFRESADNELKGILSVCDEPLVSVDFRCSDVSSTVDSSLTLVMGDDMVKVIAWYDNEWGYSQRVVDLADIVANNWK

>Cm073370

MAGDKRIKIGINGFGRIGRLVARVALQRDDVELVAVNDPFISTDYMTYMFKYDSVHGQWKHNELKVKDEKTLLFGEKPVAVFGFRNPEEIPWAKTGAEYVVESTGVFTDKDKAAAHLKGGAKKVVISAPSKDAPMFVVGVNEKEYKPELDIVSNASCTTNCLAPLAKVIHDKFGIVEGLMTTVHSITATQKTVDGPSMKDWRGGRAASFNIIPSSTGAAKAVGKVLPALNGKLTGMSFRVPTVDVSVVDLTVRLEKEATYEEIKNAIKEESEGKLKGILGYTEEDVVSTDFVGDSRSSIFDAKAGIALSKNFVKLVSWYDNEWGYSSRVIDLIVHMAKTQA

> Cm265950

MAFSSLFRSTASASLVRADLTSSPSDRVKGSSTAAFSRNLNTSSIFGTSVPSGSSSSSLQTCAAKSIQPIRATATEIPPTIQKPRSDGNTKVGINGFGRIGRLVLRVAAFRDDVDVVAVNDPFIDAKYMAYMFKYDSTHGVFKGTINVVDDSTLEINGKLIKVFSKRDPAEIPWGDYGVDYVVESSGVFTTIAKASAHMKGGAKKVVISAPSADAPMFVVGVNEKTYKPNMNIVSNASCTTNCLAPLAKVVHEEFGILEGLMTTVHATTATQKTVDGPSMKDWRGGRGASQNIIPSSTGAAKAVGKVLPDLNGKLTGMAFRVPTANVSVVDLTCRLAKSASYEDVKAAIKYASEGSLKGILGYTDEDVVSNDFVGDSRSSIFDAKAGIGLSASFMKLVSWYDNEWGYSNRVLDLIEHMALVAARN

>Cm128480

MLEISLHVMVMVDLTERLEKKATYDDIKAAHKEASQGEMKGIFGYTEDDVVSTDFVGDNRSSIFDAEAGISLSDNFVKLVSWYDTEWSYSTGGIGGDWRMQTSTRERMHLQETVNFTVSATLVFKSPLIT

>Cm128490

MFVMGVNEKEYKKELDIVSNVSCTTTCLASLAKVIQNKFGIVEGFMTNVHAIAGDYGHIWQAPVATAVNAIV

> Cm063290

MLFFIFTITKRSSIFYAKAGIALSKNFVKLVSWYDNEWGYSSRVIDLIVHMARTQA*

> Cm309190

MHAKFERVLLNIGNPERTLKETAACAAVLSSIFYAKAGIALSENFVKLVSWYDNEWGYSSRVII

>Ci039800

MAFSSLLRSTASASLVRADLTSSPSDRVKGSSTVAFSRNLNTSSIFGTSVPSGSSSSSLQTCAAKSIQPIRATATEIPPTIQKPRSDGNTKVGINGFGRIGRLVLRVAAFRDDVDVVAVNDPFIDAKYMAYMFKYDSTHGVFKGTINVVDDSTLEINGKLIKVFSKRDPAEIPWGDYGVDYVVESSGVFTTIAKASAHMKGGAKKVVISAPSADAPMFVVGVNEKTYKPNMNIVSNASCTTNCLAPLAKVVHEEFGILEGLMTTVHATTATQKTVDGPSMKDWRGGRGASQNIIPSSTGAAKAVGKVLPDLNGKLTGMAFRVPTPNVSVVDLTCRLAKGASYEDVKAAIKYASEGSLKGILGYTDEDVVSNDFVGDSRSSIFDAKAGIGLSASFMKLVSWYDNEWGYSNRVLDLIEHMALVAAHN*

>Ci152630

MAGDKKIKIGINGFGRIGRLVARVALQRDDVELVAVNDPFISTDYMTYMFKYDSVHGQWKHNELKVKDEKTLLFGEKPVAVFGFRNPEEIPWAKTGAEYVVESTGVFTDKDKAAAHLKGGAKKVVISAPSKDAPMFVVGVNEKEYKPELDIVSNASCTTNCLAPLAKVIHDKFGIVEGLMTTVHSITATQKTVDGPSMKDWRGGRAASFNIIPSSTGAAKAVGKVLPALNGKLTGMSFRVPTVDVSVVDLTVRLEKEATYEEIKNAIKEESEGKLKGILGYTEEDVVSTDFVGDSRSSIFDAKAGIALSKNFVKLVSWYDNEWGYR

>Ci185410

MASHSALAPSRIPAITRIPSKTTHSFPTQCSTKRLDVAEFAGLRANAGATYATGARDASFFDAVTAQLTPKVAAGSVPIKKETVAKLKVAINGFGRIGRNFLRCWHGRKDSPLDVVVVNDSGGVKNASHLLKYDSLLGTFKADVKIVDNETISVDGKLIKVVSNRDPLQLPWAELGIDIVIEGTGVFVDGPGAGKHIQAGAKKVIITAPAKGADIPTYVVGVNEKDYDHEVANIVSNASCTTNCLAPFVKVMDEELGIVKGAMTTTHSYTGDQRLLDASHRDLRRARAAALNIVPTSTGAAKAVSLVLPQLKGKLNGIALRVPTPNVSVVDLVVNVEKKGITAEDVNAAFRKAAEGPLKGILAVCDVPLVSVDFRCSDVSSTIDSSLTMVMGDDMVKVVAWYDNEWGYSQRVVDLAHLVATKWPGVAAGGSGDPLEDFCQTNPADEECKVYEA

>Ci239530

MASATLSVAKSALQGNGKGFSEFSGLRNSASLPFGRKSSDDFHSVIALQTSALGSSSSGYRKVAAQAKLKVAINGFGRIGRNFLRCWHGRKDSPLEVVAINDTGGVKQASHLLKYDSTLGIFEADVKPVGTDGISVDGKVIQVVSNRNPVNLPWGDLGIDLVIEGTGVFVDREGAGKHIQAGAKKVLITAPGKGDIPTYVVGVNADAYKPDEPIISNASCTTNCLAPFVKVLDQKFGIIKGTMTTTHSYTGDQRLLDASHRDLRRARAAALNIVPTSTGAAKAVALVLPALKGKLNGIALRVPTPNVSVVDLVVQVSKKTFAEEVNAAFRESADNELKGILSVCDEPLVSVDFRCSDVSSTVDSSLTLVMGDDMVKVIAWYDNEWGYSQRVVDLADIVANNWK

>Ci248760

MTYMFKYDSVHGQWKHHELKVKDDKTLLFGEKPVTVFGVRNPEEIPWAETGAEYVVESTGVFTDKDKAAAHLKGGAKKVIISAPSKDAPMFVVGVNENEYKPELNIVSNASCTTNCLAPLAKVIHDKFGIVEGLMTTVHSITATQKTVDGPSSKDWRGGRAASFNIIPSSTGAAKAVGKVLPALNGKLTGMAFRVPTVDVSVVDLTVRLEKDASYDEIKAAIKAESEGKLKGILGYTEDDVVSTDFVGDSRSSIFDAKAGIALSKKFVKLVSWYDNEWGYSTRVVDLIVHMSKTQ

>Ci075420

MIKEGIDISDDDSPAMKIFSTYMFKYDSVRGQWKHQLKVKDSTTLLFGEKPDTVFVNPEEIPWAETGAELAVESTGVFTDKDKAAAHLKGGTKKVVISAPSKDAPMFVMGVNEKEYKKELDIVSNASCTTTCLASLAKVIQNKFGIVEGFMTTVHAIAATQKTVDGPSMKDWRGGRAASFNIIPSSDGAAKEYIAVGKVLPSLNDKLTGMAFRLPRQCLSGRPHCEMKGIFCYTEDDVVSTDFVGDNRSSIFYAKAGISLSDNFEKLVSWDWRLENAD

>Ci310010

MFKYDSVRGQWKHQLKVKDSTTLLFGEKPDTVFVNPEEIPWAETGAELAVESTGVFTDKDKAAAHLKGGTKKVVISAPSKDAPMFVMGVNEKEYKKELDIVSNASCTTTCLASLAKVIQNKFGIVEGFMTTVHAIAATQKTVDGPSMKDWRGGRAASFNIIPSSDGAAKEYIAVGKVLPSLNGKLTGMAFRLPRQCLSGRPHCETREESHLR

> Ci310500

MFKYDSVRGQWKHQLKVKDSTTLLFGEKPDTTGAELAVESTGVFTDKDKAAAHLKGGTKKVVISAPSKDAPMFVMGVNEKEYKKELDIVSNASCTTTCLASLAKVIQNKFGIVEGFMTTVHAIA

>Ci183330

MKLPARFDKRVLLNIGNPERTLRETAACAAVSFSHLPPPTSSIFYAKAGIALSKNFVKLVSWYDNEWGYSSRVIDLIVHMARTQA

> Ci243110

MAPERDIGGINNNGSVKIHEACKVPPFSDDSTAAAVGATTLPLTYFDTYCFNLSPVDILLFYEIKDLTWDSFNSEILPKLKHSLSITLLHYLPLAGHLMWPPDAEKPAVCYFKDDGVSVTVAESNADLRLLSSNNGTRRAVEFHPLAPRLSISDDKAEVTAIQITLFPNQGFSICISVHHTVIDGKSAVLFIKSWAYLCKKLQQQNVVVPVPSLLPGSLTPCFDRSLIKDPRGIDMVYVKQLMAFTGSDPNTRSLKVTIPVTKVDSNLVRKTFTLTREDLNKLKHKLVLVNKDQHPSKQLHLSTFVLTCAHVFVCLVKARGEDANTTVTFWVPADCRSRLDPPLPGNYFGSCILAEFAVAKAGDFMQEDGIAFVAEKLSDSVKGLKKGDAIIGTEDRIARELEVMKETGEQSIQIDIAGGTNHFDFYDSDFGWGKPNKVEIVSIDGTGAISLMQSRDGGSVEIGVVLDEPQMEVFTSLFVSGLNDRENTSHPLRTVLRVLLNIGNPERTLRETAACAAVSFSHLPPPTSSIFYAKASIALSKNFVKLVSWYDNEWGYSSRVIDLIVHMAKTQA

>sb27156

MAFSSLLRSTASVSLVRADLTSSPSDRVKGSSTVAFSRNLNTSSIFGTSVPSGSSSSSLQTCSAKSIQPIRATATEIPPTIQKSRSDGNTKVGINGFGRIGRLVLRVAAFRDDVDIVAVNDPFIDAKYMAYMFKYDSTHGVFKGTINVVDDSTLEINGKQIKVFSKRDPAEIPWGDYGVDYVVESSGVFTTIAKASAHMKGGAKKVVISAPSTDAPMFVVGVNEKTYKPNMNIVSNASCTTNCLAPLAKVVHEEFGILEGLMTTVHATTATQKTVDGPSMKDWRGGRGASQNIIPSSTGAAKAVGKVLPDLNGKLTGMAFRVPTPNVSVVDLTCRLAKSASYENVKAAIKYASEGSLKGILGYTDEDVVSNDFVGDSRSSIFDAKAGIGLSASFMKLVSWYDNEWGYSNRVLDLIEHMALVAAHN

>sb10897

MGKVKIGINGFGRIGRLVARVILQRDDVELVAVNDPFITTDYMTYMFKYDSVHGQWKHHELKVKDDKTLLFGEKPVTVFGIRNPEEIPWAETGAEYVVESTGVFTDKDKAAAHLKGGAKKVIISAPSKDAPMFVVGVNEHEYKPEHNIVSNASCTTNCLAPLAKVIHDKFGIVEGLMTTVHSITATQKTVDGPSSKDWRGGRAASFNIIPSSTGAAKAVGKVLPALNGKLTGMAFRVPTVDVSVVDLTVRLEKDASYDEIKAAIKAESEGKLKGILGYTEDDVVSTDFVGDSRSSIFDAKAGIALSKKFVKVVSWYDNEWGYSTRVVDLIVHMSKTQ

>sb35733

MASHSALAPSRIPAITRIPSKTTHSFPTQCSTKRLDVAEFAGLRANAGATYATGARDASFFDAVTAQLTPKVAAGSVPVKKETVAKLKVAINGFGRIGRNFLRCWHGRKDSPLDVVVVNDSGGVKNASHLLKYDSMLGTFKADVKIVDNETISVDGKLIKVVSNRDPLRLPWAELGIDIVIEGTGVFVDGPGAGKHIQAGAKKVIITAPAKGADIPTYVVGVNEKDYDHQASNIVSNASCTTNCLAPFVKVMDEELGIVKGTMTTTHSYTGDQRLLDASHRDLRRARAAALNIVPTSTGAAKAVSLVLPQLKGKLNGIALRVPTPNVSVVDLVVNVEKKGITAEDVNAAFRKAAGGPLKGILAVCDVPLVSVDFRCSDVSSTIDSSLTMVMGDDMVKVVAWYDNEWGYSQRVVDLAHLVASKWPGVAAGGSGDPLEDFCQTNPADEECKVYEA

>sb27073

MASATLSVAKSALQGNGKGFSEFSGLRNSASLPFGRKSSDDFHSVIALQTSALGSSSSGYRKVAAQAKLKVAINGFGRIGRNFLRCWHGRKDSPLEIVAINDTGGVKQASHLLKYDSTLGIFDADVKPVGTDGISVDGKVIQVVSNRNPVNLPWGDLGIDLVIEGTGVFVDREGAGKHIQAGAKKVLITAPGKGDIPTYVVGVNADAYNPDEPIISNASCTTNCLAPFVKVLDQKFGIIKGTMTTTHSYTGDQRLLDASHRDLRRARAAALNIVPTSTGAAKAVALVLPALKGKLNGIALRVPTPNVSVVDLVVQVSKKTFAEEVNAAFRESSDNELKGILSVCDEPLVSVDFRCSDVSSTVDSSLTLVMGDDMVKVIAWYDNEWGYSQRVVDLADIVANNWK

>sb15342

MTYMFKYDSVHGQWKHNELKVKDEKTLLFGEKPVAVFGFRNPEEIPWAKTGAEFVVESTGVFTDKDKAAAHLKGGAKKVVISAPSKDAPMFVVGVNEKEYKPELDIVSNASCTTNCLAPLAKVIHDKFGIVEGLMTTVHSITATQKTVDGPSMKDWRGGRAASFNIIPSSTGAAKAVGKVLPALNGKLTGMSFRVPTVDVSVVDLTVRLEKEATYEDIKNAIKEQSEGKLKGILGYTEEDVVSTDFVGDSRSSIFDAKAGIALSKNFVKLVSWYDNEWGYRFN

>sb26627

MYVMGVNGKEYNKELDIVSNASCTTTCLAPLAKVIHNKFGIVEGLMTTVHAITATQKTVDGPSMKDWRGGRAASFNIIPSSTGAAKVLGKVLPTLNGKLTGMDFRVPTVDVSVVNLTVRPEKKATYDDIKAAIKQLIKHYHHFLN

> sb26598

MGKKIKIGTNGFGRISRLAAKVALQRDDVELVAVNDPFITTDYINPEEIPWVESGAEFVVESTGAFTDKDKAAAL
